# Ketosis prevents abdominal aortic aneurysm rupture through C–C chemokine receptor type 2 downregulation and enhanced extracellular matrix balance

**DOI:** 10.1038/s41598-024-51996-7

**Published:** 2024-01-16

**Authors:** Sergio Sastriques-Dunlop, Santiago Elizondo-Benedetto, Batool Arif, Rodrigo Meade, Mohamed S. Zaghloul, Hannah Luehmann, Gyu S. Heo, Sean J. English, Yongjian Liu, Mohamed A. Zayed

**Affiliations:** 1 Section of Vascular Surgery, Department of Surgery, Washington University School of Medicine, St. Louis, MO USA; 2 Department of Radiology, Washington University School of Medicine, St. Louis, MO USA; 3 Division of Molecular Cell Biology, Washington University School of Medicine, St. Louis, MO USA; 4 Department of Biomedical Engineering, McKelvey School of Engineering, Washington University, St. Louis, MO USA; 5 Veterans Affairs St. Louis Health Care System, St. Louis, MO USA

**Keywords:** Inflammation, Cardiovascular diseases, Vascular diseases, Aneurysm

## Abstract

Abdominal aortic aneurysms (AAAs) are prevalent with aging, and AAA rupture is associated with increased mortality. There is currently no effective medical therapy to prevent AAA rupture. The monocyte chemoattractant protein (MCP-1)/C–C chemokine receptor type 2 (CCR2) axis critically regulates AAA inflammation, matrix-metalloproteinase (MMP) production, and extracellular matrix (ECM) stability. We therefore hypothesized that a diet intervention that can modulate CCR2 axis may therapeutically impact AAA risk of rupture. Since ketone bodies (KBs) can trigger repair mechanisms in response to inflammation, we evaluated whether systemic ketosis in vivo could reduce CCR2 and AAA progression. Male Sprague–Dawley rats underwent surgical AAA formation using porcine pancreatic elastase and received daily β-aminopropionitrile to promote AAA rupture. Rats with AAAs received either a standard diet, ketogenic diet (KD), or exogenous KBs (EKB). Rats receiving KD and EKB reached a state of ketosis and had significant reduction in AAA expansion and incidence of rupture. Ketosis also led to significantly reduced aortic CCR2 content, improved MMP balance, and reduced ECM degradation. Consistent with these findings, we also observed that *Ccr2*−/− mice have significantly reduced AAA expansion and rupture. In summary, this study demonstrates that CCR2 is essential for AAA expansion, and that its modulation with ketosis can reduce AAA pathology. This provides an impetus for future clinical studies that will evaluate the impact of ketosis on human AAA disease.

## Introduction

Abdominal aortic aneurysm (AAA) formation, expansion, and subsequent rupture results from a complex series of biomolecular processes^1,2^. AAAs are often asymptomatic during their formation and expansion stages, but lead to a high risk of morbidity and mortality when they spontaneously rupture^3,4^. Unfortunately, there is currently no effective medical therapy to alleviate AAA expansion and the risk of rupture. Invasive surgery is the only available management for AAAs that meet the traditional aortic diameter criteria or are rapidly expanding in diameter^5^. Given the limited medical treatment options for individuals with small AAAs that do not yet meet criteria for surgical repair, expectant management is usually the only remaining option^6^. Therefore, clinical stabilization of small AAAs remains a large unmet need, and a longer-term management strategy for individuals with AAA disease can be a tremendous value add^7^.

Inflammation plays an essential role in AAA disease progression and risk of rupture^3^. The release of inflammatory mediators within the aortic wall leads to a cascade of biomolecular signals that lead to the activation of matrix metalloproteinases (MMPs) that consequentially lead to extracellular matrix (ECM) degradation^8–11^. The C–C chemokine receptor type 2 (CCR2) mediates trafficking of leukocytes to site of aortic tissue inflammation following initial and repeated injury^12^. Our team previously demonstrated that CCR2 content in AAAs, as demonstrated by positron emission tomography (PET)/computed tomography (CT) imaging in a preclinical rodent model, highly correlates with the incidence of AAA expansion and the risk of rupture^13,14^. However, it remains unknown whether CCR2 content is essential for AAA rupture, and whether modulation of CCR2 can alleviate disease progression.

Various dietary interventions can impact immune function and inflammation^15^. Ketogenesis in particular can dramatically impact anti-inflammatory signaling, and is reported to promote vascular tissue repair^15–17^. As a natural physiologic process that leads to the production of ketone bodies (KBs) such as acetoacetate (AcAc), beta-hydroxybutyrate (βHB) and acetone, ketogenesis not only serves as an alternative fuel source for organ systems, but it also activates signaling cascades that can impact various cell functions. Although high fat diets are linked to increased AAA expansion and aortic plaque formation^18,19^, recent studies suggested that a high fat—low carbohydrate ketogenic diet, as well as exogenous ketone body supplementation, can reduce tissue inflammation and ameliorate the risk of vascular injury and atheroprogression due to increased catabolic metabolism^20,21^. It is unknown whether these potential benefits are limited to atherosclerosis, or whether ketosis can also have a broader impact on degenerative aortopathies such as AAAs. Therefore, we hypothesized that nutritional ketosis, either in the form of a ketogenic diet (KD) or exogenous ketone body (EKB) supplementation, can impact CCR2-mediated inflammation and improve MMP balance in aortic tissue to reduce the risk of AAA progression, aortic wall inflammation, ECM content, and the incidence of AAA rupture.

## Results

### Ketosis attenuates AAA formation and content of MMP9 in rat aortic tissue

**Figure 1:**
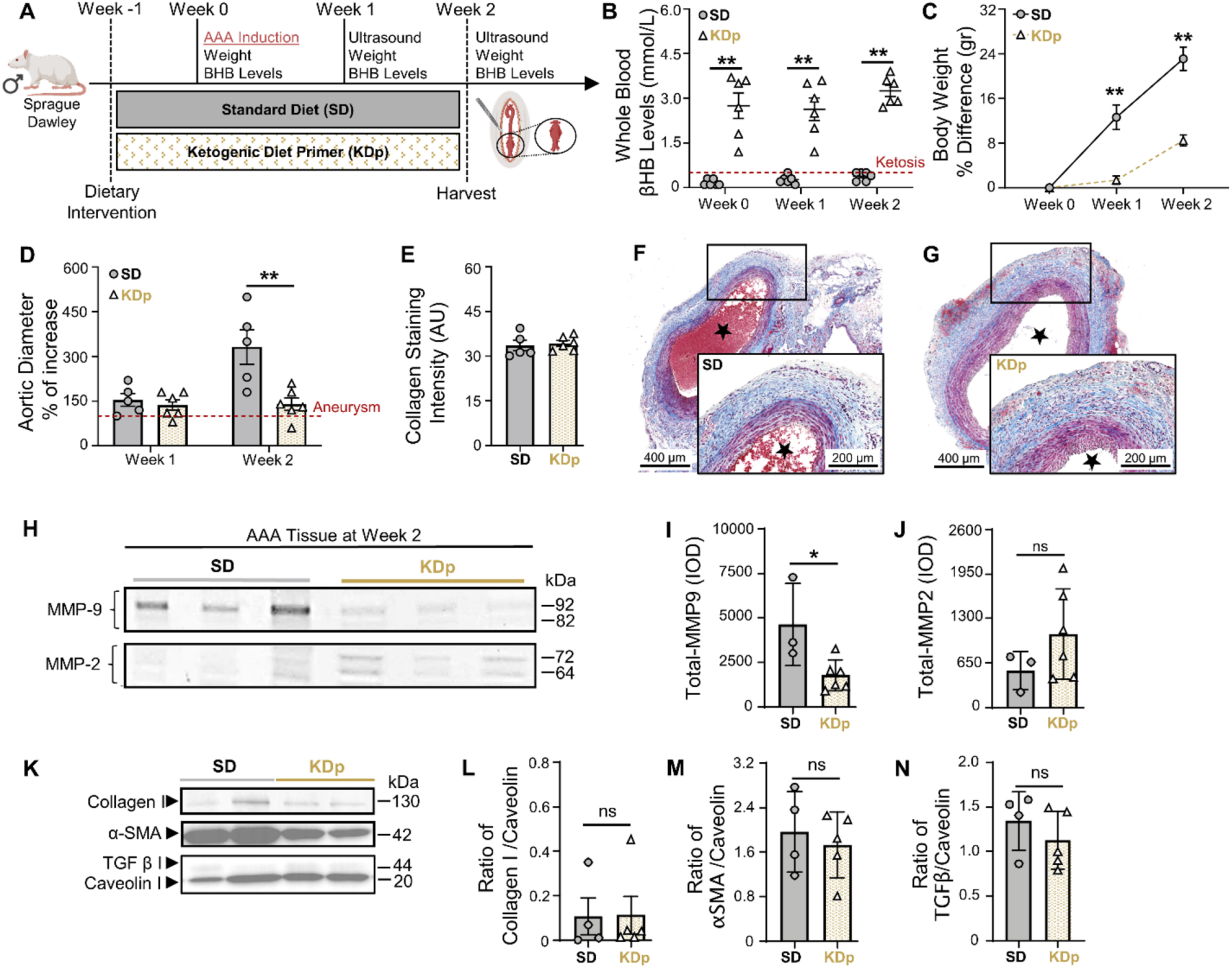
Ketosis attenuates AAA formation and MMP9. (**A**) Rats underwent exposure to PPE for AAA creation. The experimental group was given a ketogenic diet that started one-week prior to PPE exposure (KDp; N = 6) while the control group was fed a standard chow diet (SD; N = 5). (**B**) Ketosis (βHB whole blood levels > 0.5 mM/L) was verified at week 0, 1 and 2 in SD (0.2 ± 0.1, 0.3 ± 0.1 and 0.4 ± 0.1) and KDp rats (3 ± 1, 3 ± 1 and 3 ± 0.5) respectively (*p* < 0.01). (**C**) Percent body weight difference in SD versus KDp rats at week 1 (13 ± 5 vs. 2 ± 1.3) and at week 2 (23 ± 5 vs. 8 ± 2) respectively (*p* < 0.001). (**D**) Percent aortic diameter in SD versus KDp rats at week 1 (154 ± 48 vs. 137 ± 42; *p* = ns) and at week 2 (332 ± 129 vs. 140 ± 152; *p* = 0.008) respectively (aneurysms were defined by a > 100% increase in the aortic diameter compared with pretreatment measurements). (**E**) AAA collagen staining quantification for SD and KDp at week 2 (33 ± 4 vs. 34 ± 2; *p* = ns) respectively. (**F** and **G**) Trichrome staining of abdominal aortas (cross-section of tissue slides) with 5× magnification for SD and KDp rats. Areas with blue staining signify areas with higher collagen deposition. (**H**) Zymogram demonstrating total MMP9 and MMP2 levels were measured by integrated optical density (IOD). (**I**) Total MMP-9 levels for SD and KDp at week 2 (4.6 ± 2 × 10^3^ vs. 1.7 ± 0.8 × 10^3^; *p* = 0.02) respectively. (**J**) Total MMP-2 levels for SD and KDp at week 2 (5.4 ± 3 × 10^2^ vs. 1 ± 0.6 × 10^3^; *p* = ns) respectively. (**K**) Representative western blots of collagen 1, α-SMA, TGFβ-1 and Caveolin 1 in AAA tissue. (**L**) Collagen I protein content expressed as a ratio to Caveolin 1 content in AAA tissue of SD and KDp rats (0.1 ± 0.1 vs. 0.1 ± 0.1 respectively; *p* = ns). (**M**) α-SMA protein content expressed as a ratio to Caveolin 1 content in AAA tissue of SD and KDp rats (2 ± 0.7 vs. 1.7 ± 0.6 respectively; *p* = ns). (**N**) TGFβ protein content expressed as a ratio to Caveolin 1 content in AAA tissue of SD and KDp rats (1.3 ± 0.3 vs. 1.2 ± 0.3 respectively; *p* = ns). Data presented as mean ± standard deviation. ns > 0.05, *p < 0.05, **p < 0.01, ***p < 0.001 using Student’s t test.

We evaluated AAA pathology in rats maintained on either ketogenic diet (KD) or standard diet (SD). Rats maintained on a ketogenic diet prior to AAA induction (KDp; Fig. 1A) achieved a state of sustained ketosis from day 0–14 (Fig. 1B), and as previously reported^22^, had a moderate decrease in weight gain by week 1 (*p* < 0.001; Fig. 1C). By week 2 there was a substantial 42% decrease in AAA diameter in KDp rats (*p* = 0.008; Fig. 1D and Supplementary Fig. S1A). Aortic wall media demonstrated equivalent mason trichrome (MT)-stained collagen between rats maintained on SD and KDp (Fig. 1E–G). Zymography analysis of harvested aortic tissue at week 2 demonstrated a significant decrease in total-MMP9 in KDp rats whereas MMP2 was unchanged (Fig. 1H–J). Western blot of type 1 Collagen, α-smooth muscle actin (α-SMA) and TGF-β content demonstrated comparable values between KDp and SD rats (Fig. 1K–N). These data suggested that diet-induced ketosis can inhibit AAA expansion, and that this may in part be due to a decrease in aortic wall total MMP9 without substantial modification of the aortic architecture.

### Sustained ketosis reduces AAA expansion and CCR2 uptake in rupture-prone rats

**Figure 2:**
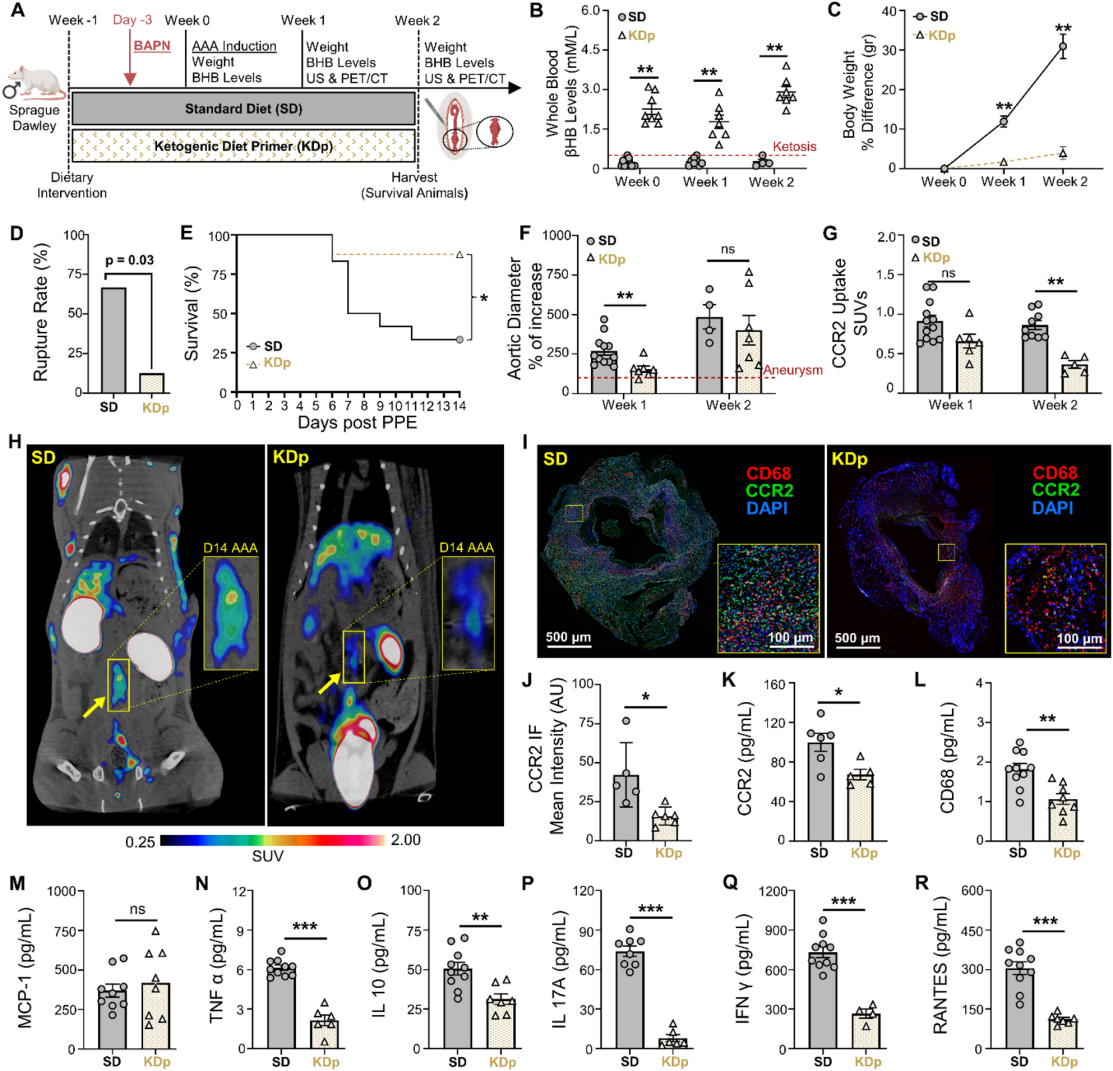
Sustained ketosis reduces AAA expansion and risk of rupture via CCR2 downregulation and Collagen 1 preservation. (**A**) Rats underwent exposure to PPE to develop AAAs and were also treated with β-aminoprionitrile (BAPN) to promote AAA rupture. (**B**) Ketosis (βHB whole blood levels > 0.5 mM/L) in SD and KDp rats at week 0 (0.2 ± 0.1 vs. 2 ± 0.5), week 1 (0.3 ± 0.1 vs. 1.8 ± 0.7) and week 2 (0.2 ± 0.1 vs. 3 ± 0.5; *p* < 0.01). (**C**) Percent body weight difference in SD and KDp rats at week 1 (12 ± 5 vs. 2 ± 1.3; *p* < 0.001) and week 2 (31 ± 6 vs. 4 ± 3; *p* = 0.006). (**D**) AAA rupture event rate with statistical analysis in SD and KDp rats (*p* = 0.03). (**E**) Kaplan–Meier curve demonstrating rate of survival following BAPN administration. 67% (8/12) of SD rats and 12% (1/8) KDp rats developed AAA rupture. (**F**) Percent aortic diameter in SD and KDp rats at week 1 (270 ± 93 vs. 154 ± 53; *p* = 0.002) and week 2 (485 ± 153 vs. 401 ± 246; *p* = ns). (**G**) Quantitative tracer uptake of CCR2 content in AAA tissue for SD and KDp rats at week 1 (0.9 ± 0.2 vs. 0.7 ± 0.2; *p* = 0.05) and week 2 (0.9 ± 0.2 vs. 0.4 ± 0.1; *p* < 0.001. (**H**) Representative PET/CT coronal images at day 14 post PPE exposure showed specific and intensive detection of AAA (yellow rectangle) in SD, compared with the low trace accumulations in the KDp group of rats. (**I**) Immunofluorescence staining of abdominal aortas (cross-sectional; 5× magnification and 10× magnification) marked with CCR2 (in green: CCR2 + cells) and CD68 marker (in red: CD68+ cells; macrophages) to visualize inflammatory cells infiltration within the AAA. (K) CCR2 concentration at week 1 in AAA tissue of SD and KDp rats (100 ± 22 vs. 67 ± 12; *p* = 0.02). (**K**) Macrophage marker CD68 content at week 1 in AAA tissue of SD and KDp rats (1.8 ± 0.4 vs. 1 ± 0.4; *p* = 0.002). (**L**) Chemokine MCP-1 content (3.7 ± 1.2 × 10^2^ vs. 4.2 ± 2.2 × 10^2^; *p* = ns). (**M**) Pro-inflammatory marker TNFα content (6.1 ± 0.6 vs. 2.1 ± 1; *p* = 0.001), (**N**) IL-10 content (5 ± 1.3 × 10^1^ vs. 3.1 ± 0.9 × 10^1^; *p* = 0.03), (**O**) IL-17A content (7.4 ± 1.1 × 10^1^ vs. 0.8 ± 0.6 × 10^1^; *p* < 0.001), (**P**) IFNγ content (7.3 ± 1.3 × 10^2^ vs. 2.6 ± 0.7 × 10^2^; *p* = 0.002), (**Q**) RANTES content (3 ± 0.7 × 10^2^ vs. 1.1 ± 0.2 × 10^2^; *p* < 0.001). Data presented as mean ± standard deviation. ns > 0.05, *p < 0.05, **p < 0.01, ***p < 0.001 using Student’s t test.

The risk of AAA progression correlates with CCR2-mediated pro-inflammatory signaling^14,23–25^. We therefore evaluated whether dietary ketosis could impact CCR2 content using a AAA rupture model. For this we evaluated a cohort of rats that received either SD or KDp after AAA induction with PPE, as well as daily β aminopropionitrile (BAPN) administration to promote AAA rupture (Fig. 2A). Rats fed KDp, and received daily BAPN, remained in a state of ketosis from days 0–14 (Fig. 2B and Supplementary Fig. S2B), and weight gain was reduced at weeks 1 and 2 (*p* < 0.001 and *p* = 0.006, respectively; Fig. 2C and Supplementary Fig. S2C). Administration of BAPN did not significantly alter βHB levels between SD and KDp rats (Supplementary Fig. S3A). Notably, KDp rats also had significantly reduced AAA rupture rate (67% vs. 12%; *p* = 0.03; Fig. 2D and E), and aneurysm diameter at week 1 was significantly decreased in KDp rats (*p* = 0.002 and *p* = 0.01; Fig. 2F and Supplementary Fig. S1B). Although by week 2, AAA diameter was equivalent between groups, PET/CT with ^64^Cu-DOTA-ECL1i (selective CCR2-targeting PET radiotracer^12,14,26^) demonstrated significantly reduced CCR2 uptake in AAAs of KDp rats throughout study period (week 1, *p* = 0.05; week 2, *p* =  < 0.0001; Fig. 2G and H). ^18^F-fluorodeoxyglucose PET/CT performed in KDp and SD rats at week 1 revealed comparable AAA signal uptake^14,27,28^, (*p* = ns; Supplementary Fig. S4A and B). These findings demonstrate that KDp rats developed smaller aneurysms, had a combined 54% absolute risk reduction in AAA rupture, and decreased CCR2 uptake in AAA tissue.

### Sustained ketosis inhibits cytokine profiles downstream to CCR2 in AAA tissue by week 1

We next evaluated whether sustained ketosis impacts CCR2 in AAA tissue and downstream cytokine profiles during AAA formation. We observed that immunostaining of AAA tissue in KDp rats demonstrated a marked decrease in CCR2 and CD68+ macrophages (Fig. 2I and J; Supplementary Figs. S5 and S6). KDp rats had reduced CD86+ pro-inflammatory macrophages (M1 phenotype; *p* = 0.04), whilst a modest increase in CD206+ pro-regenerative macrophages (M2 phenotype; *p* = ns), within the AAA wall (Supplementary Fig. S7A–D). Similarly, by using ELISA we confirmed that CCR2 and CD68 concentration was significantly decreased in AAAs of KDp rats at week 1 (*p* = 0.02 and *p* = 0.002, respectively; Fig. 2K and L). The CCR2 ligand, monocyte chemoattractant protein-1 (MCP-1) was unchanged between KDp and SD rats (Fig. 2M), but CCR2-dependent pro-inflammatory cytokines TNFα, IL-10, IL-17A, and IFN γ were significantly decreased in AAA tissue of KDp rats (*p* = 0.001, *p* = 0.03, *p* < 0.001, and *p* = 0.002 respectively; Fig. 2N–Q). Similarly, RANTES (the ligand for C–C motif chemokine receptor 5; CCR5) was significantly reduced in KDp rats (*p* < 0.001; Fig. 2R). These results indicates that KDp notably decreases AAA pro-inflammatory macrophage infiltration, CCR2-dependent cytokine production, and reduced AAA expansion and rupture.

### Ketosis alters AAA collagen and elastin content and MMP balance

**Figure 3:**
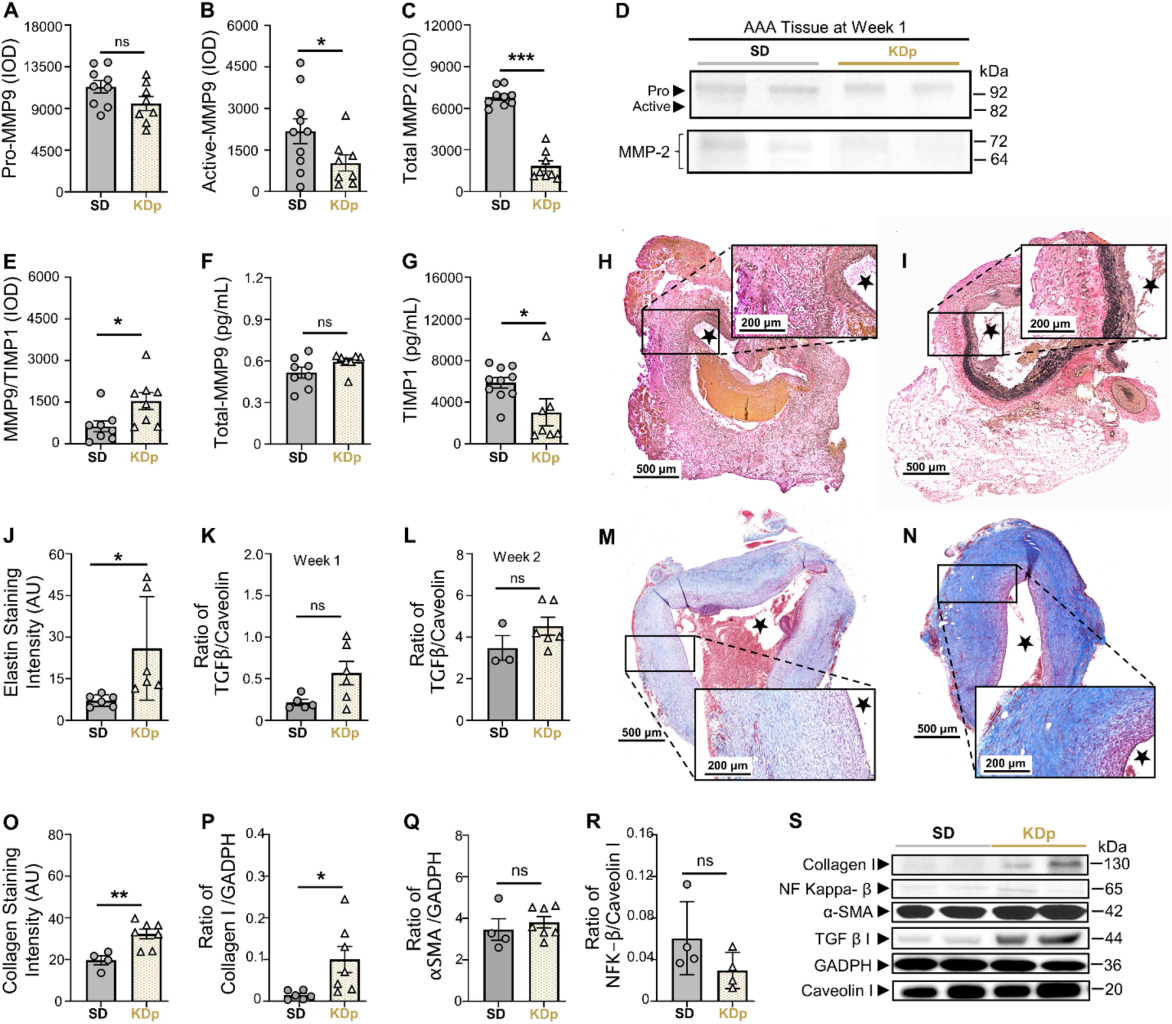
Sustained ketosis downregulates CCR2 content and inhibit its downstream signals. (**A**) Pro MMP9 levels at week 1 in AAA tissue of SD and KDp rats (11.3 ± 2 × 10^3^ vs. 9.5 ± 2.1 × 10^3^; *p* = ns). (**B**) Active MMP9 levels in AAA tissue of SD and KDp rats (2.1 ± 1.4 × 10^3^ vs. 0.9 ± 0.7 × 10^3^; *p* = 0.04). (**C**) Total MMP2 levels in AAA tissue of SD and KDp rats (7 ± 0.7 × 10^3^ vs. 1.8 ± 1 × 10^3^; *p* < 0.001). Pro and active MMP9 and total MMP2 levels were measured by integrated optical density (IOD). (**D**) Representative zymogram from AAA tissue homogenates at week 1 demonstrating pro and active MMP-9 and total MMP-2 levels in SD and KDp rats (**E**) MMP9/TIMP1 complex levels in AAA tissue of SD and KDp rats (6.3 ± 5 × 10^2^ vs. 15 ± 8.7 × 10^2^; *p* = 0.02). ELISA of AAA tissue homogenates in SD and KDp rats provided levels of (**G**) Total MMP9 (5 ± 1.1 × 10^–1^ vs. 6 ± 0.6 × 10^–1^; *p* = ns), (**H**) SD AAA tissue harvested at day 6 (week 1) stained with VVG at 10× and 5× magnification respectively. TIMP 1 (5.9 ± 1.6 × 10^3^ vs. 3.4 ± 3 × 10^3^; *p* = 0.03). (**I**) KD AAA tissue harvested at day 6 (week 1) stained with VVG at 10× and 5× magnification respectively. (**J**) AAA VVG-staining quantification for SD and KDp at week 1 (7 ± 2 vs. 26 ± 18 respectively; *p* = 0.03). (**K**) TGFβ-1 protein content expressed as a ratio to Caveolin 1 content in AAA tissue from SD and KDp rats at week 1 (2.2 ± 0.8 × 10^–1^ vs. 5.7 ± 3.4 × 10^–1^; *p* = 0.2). (**L**) TGFβ-1 protein content expressed as a ratio to Caveolin 1 content in AAA tissue from SD and KDp rats at week 2 (0.3 ± 0.1 vs. 0.5 ± 0.1; *p* = ns). (**M** and **N**) Trichrome staining of abdominal aortas (cross-sectional) with 5× magnification and 10× magnification in SD and KDp rats to visualize collagen deposition in animal aortic tissue. (**O**) AAA collagen staining quantification for SD and KDp at week 2 (20 ± 4 vs. 32 ± 6; *p* = 0.006). (**P**) Collagen 1 protein content expressed as a ratio to GAPDH content in AAA tissue at week 2 for SD and KDp rats (1.5 ± 1.0 × 10^–2^ vs. 10 ± 8.2 × 10^–2^, *p* = 0.03). (**Q**) α-SMA protein content expressed as a ratio to GAPDH content in AAA tissue of SD and KDp rats (3.4 ± 1 vs. 3.8 ± 0.7; *p* = ns). (**R**) NF Kappa β protein content expressed as a ratio to Caveolin I content in AAA tissue of SD and KDp rats (0.06 ± 0.03 vs. 0.03 ± 0.01; *p* = ns). (**S**) Representative Western blots of collagen 1, NF Kappa β, α-SMA, TGFβ-1, GAPDH and Caveolin 1 in AAA tissue. Data presented as mean ± standard deviation. ns > 0.05, *p < 0.05, **p < 0.01, ***p < 0.001 using Student’s t test.

Previous work demonstrated that CCR2 can impact MMP balance and promote elastin degradation, while decreasing transforming growth factor beta (TGFβ) signaling that contributes to collagen formation^29^. Gelatin zymography of AAA tissue at week 1 demonstrated that although pro-MMP9 and total-MMP9 were equivalent between SD and KDp rats (Fig. 3A), active MMP9, known to promote AAA formation and rupture^30^, was significantly reduced in KDp rats (*p* = 0.04; Fig. 3B). Similarly, total MMP 2, known to promote AAA expansion^31^, was also significantly reduced in KDp rats (*p* < 0.001; Fig. 3C and D). Content of MMP9 and Tissue Inhibitor of Metalloproteinases 1 complex (MMP9/TIMP1; known to prevent MMP9 over-activation) was also significantly increased in KDp rats (*p* = 0.02; Fig. 3E; Supplementary Fig. S8A). Correspondingly, AAA tissue in KDp rats demonstrated equivalent levels of total MMP-9 (Fig. 3F), and significantly reduced TIMP1 compared to SD rats (*p* = 0.03; Fig. 3G). Overall, these data demonstrate that sustained ketosis with KDp decreases active MMP9 while increasing MMP9/TIMP1 stabilizing complex in AAA tissue.

We also observed a significant positive correlation between active MMP9 and CCR2 content in the AAA tissue in both SD and KDp rats (*p* = 0.03 and *p* = 0.39 respectively; Supplementary Fig. S8B and C). These findings suggest that CCR2 content in AAA tissue may be responsible for activating MMPs, and therefore may be contributing to a higher incidence of AAA rupture. Along with MMP attenuation, VVG-staining demonstrated significantly increased elastin staining intensity within the AAA wall media in KDp rats by week 1 (*p* = 0.03; Fig. 3H-J), suggesting that KD protects against MMP-induced elastin degradation.

Interestingly, at week 1 and 2, TGFβ content in AAA tissue in KDp rats trended higher (*p* = ns and *p* = 0.05, respectively; Fig. 3K and L), and MT-staining demonstrated significantly more collagen in the AAA media (aortic segment between internal and external elastic lamina; *p* = 0.006; Fig. 3M–O). In particular, type 1 Collagen content was increased in KDp rats compared to SD at week 2 (*p* = 0.03), while α-smooth muscle actin (αSMA) content remained unchanged (Fig. 3P and Q). Lastly, NF kappa β content in AAA tissue in KDp rats demonstrated a modest nonsignificant decrease (Fig. 3R and S).

### Impact of ketosis that is initiated ‘therapeutically’ after AAA formation

**Figure 4:**
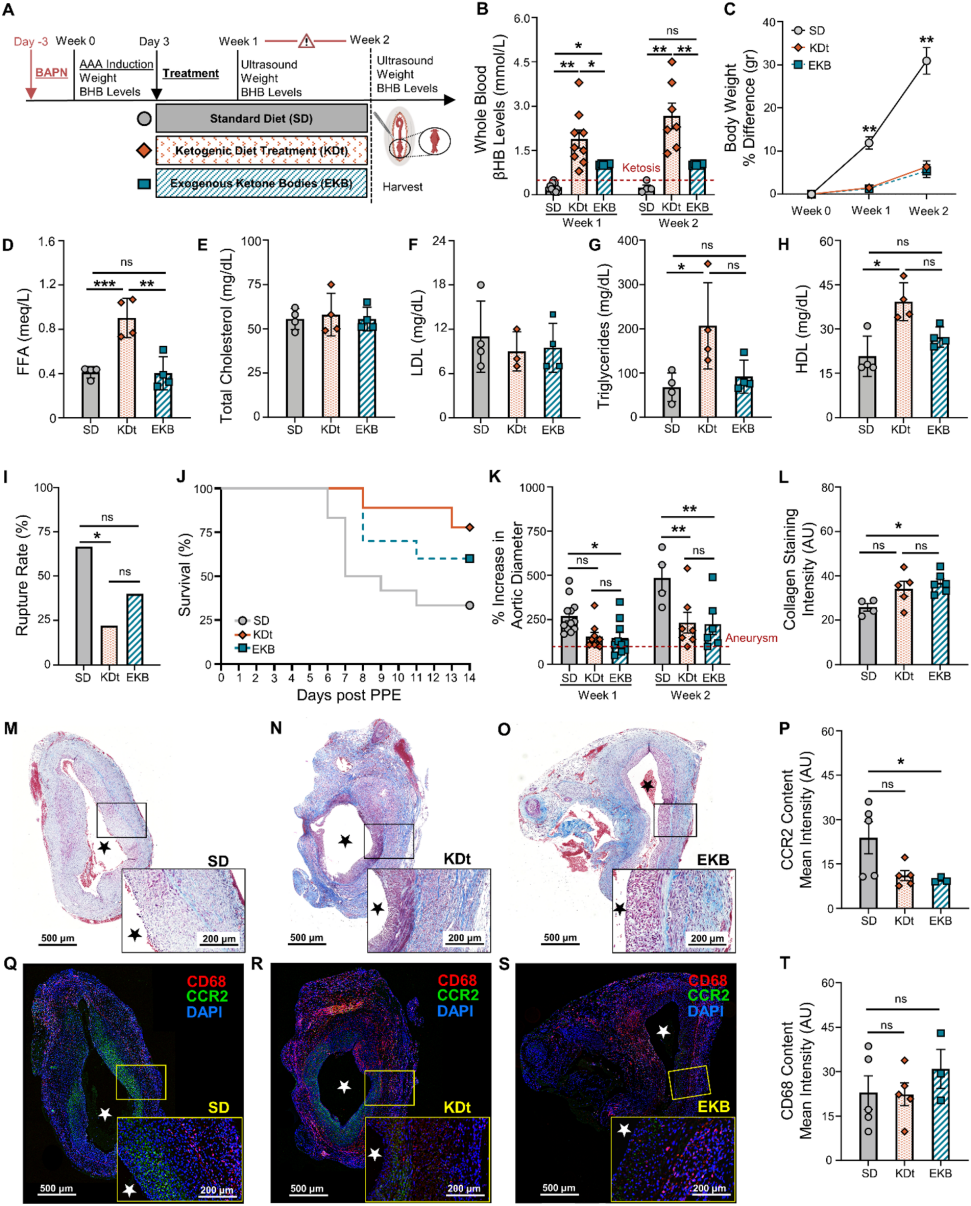
Impact of therapeutic ketosis on AAA risk of rupture. (**A**) Rats underwent exposure to PPE to develop AAAs and were also treated with BAPN to promote AAA rupture. Following AAA induction, rats received a ketogenic ‘treatment’ via an oral diet (KDt) or exogenous supplement (EKB). (**B**) Ketosis (βHB whole blood levels > 0.5 mM/L) in SD, KDt, and EKB rats at week 1 (0.2 ± 0.1, 1.8 ± 0.9 and 1 ± 0.02, respectively; *p* < 0.05) and at week 2 (0.2 ± 0.1, 2.7 ± 1.1, and 1 ± 0.02 respectively; *p* < 0.01) analyzed using two-way ANOVA. (**C**) Percent body weight difference for SD, KDt, and EKB rats at week 1 (11 ± 4, 2.5 ± 1.4, and 2.4 ± 1.3, respectively; *p* < 0.01) and at week 2 (31 ± 6, 6 ± 3.5, and 5 ± 3.6, respectively; *p* < 0.01) analyzed using two-way ANOVA. (**D**) Free fatty Acids (FFA) serum levels in meq/L in rats fed SD (0.41 ± 0.05) versus KDp (0.86 ± 0.06; *p* = 0.001), KDt (0.9 ± 0.1 *p* = 0.005) and EKB (0.40 ± 0.1; *p* = ns) respectively. (**E**) Total Cholesterol serum levels in meq/L in rats fed SD (55.5 ± 6) versus KDp (57.5 ± 10; *p* = ns), KDt (58 ± 12; *p* = ns) and EKB (55.5 ± 7; *p* = ns) respectively. (**F**) Low-density lipoprotein (LDL) serum levels in meq/L in rats fed SD (11 ± 5) versus KDp (9 ± 2; *p* = ns), KDt (9 ± 3; *p* = ns) and EKB (9.5 ± 3; *p* = ns) respectively. (**G**) Triglycerides serum levels (meq/L) in rats fed SD (68 ± 32) versus KDp (112 ± 51; *p* = ns), KDt (206 ± 97; *p* = 0.01) and EKB (92 ± 37; *p* = ns) respectively. (**H**) High-density lipoprotein (HDL) serum levels (meq/L) in rats fed SD (21 ± 7) versus KDp (26 ± 6; *p* = ns), KDt (39 ± 6; *p* = 0.01) and EKB (27 ± 3; *p* = ns) respectively. (**I**) AAA rupture event rate in SD, KDt and EKB rats (*p* < 0.05 between SD and KDt) using survival analysis. (**J**) Kaplan–Meier curve demonstrating survival following BAPN administration. 67% (8/12) of SD rats, 40% (2/9) of KDt rats (*p* = 0.03), and 22% (4/10) of EKB rats (*p* = ns) developed AAA rupture by week 2. (**K**) Percent aortic diameter at week 1 in SD versus KDt rats (270 ± 94 and 155 ± 73; *p* = 0.06), and SD versus EKB rats (148 ± 94; *p* = 0.04). At week 2, in SD versus KDt rats (485 ± 153 and 234 ± 151; *p* < 0.01), and SD versus EKB rats (227 ± 147; *p* < 0.01 analyzed using two-way ANOVA. (**L**–**O**) Trichrome staining of AAA tissue at 5× and 10× magnifications to demonstrate collagen deposition in SD versus KDt rats (26 ± 3 and 34 ± 8; *p* = ns), and SD versus EKB rats (37 ± 5; *p* = 0.02) analyzed using one-way ANOVA. (P) CCR2 ELISA content in AAA tissue of SD versus KDt rats (5.7 ± 4 and 4.6 ± 3; *p* = ns), and SD versus EKB rats (4.7 ± 3; *p* = ns). (**Q**–**S**) Immunofluorescent staining of AAA tissue at 5× and 10× magnifications to demonstrate CD68, CCR2, and DAPI positive cells. (**T**) Immunofluorescent intensity of CD68 was analyzed using one-way ANOVA between SD, KDt, and EKB groups. Data presented as mean ± standard deviation. ns > 0.05, *p < 0.05, **p < 0.01, ***p < 0.001 using one-way ANOVA, or two-way ANOVA with multiple comparison.

Rats treated with an abbreviated course of KD, therapeutically initiated 3 days post-AAA formation with PPE (KDt; Fig. 4A), also led to a state of ketosis (Fig. 4B). Rats treated with supplemental exogenous ketone bodies by oral daily gavage (EKB; Supplementary Figs. S2 and 4A) also led to ketosis, but only for 8-h per day (Fig. 4B). Like KDp rats, KDt and EKB rats also had reduced weight gain at both week 1 and 2 when compared to SD rats (*p* < 0.001; Fig. 4C and Supplementary Fig. S2).

Although KDt and EKB rats both achieved a state of ketosis throughout the study, resultant serum free fatty acids (FFA) were only significantly elevated in KDt rats when compared to EKB and SD rats (*p* = 0.005 and *p* < 0.001 respectively; Fig. 4D). While serum total cholesterol and LDL were observed to be unchanged among all rat groups (Fig. 4E and F), serum triglycerides and HDL levels were also notably elevated in KDt rats when compared to EKB and SD rats (*p* = ns and *p* < 0.05 respectively; Fig. 4G and H). These findings confirmed that KDt can lead to serum lipid changes that are expected with extended ketosis as previously demonstrated^32^.

Although, the AAA rupture rate was reduced in KDt and EKB rats compared to SD rats (40% reduction with KDt, *p* = 0.03, and 22% reduction EKB, *p* = 0.12: Fig. 4I and J), the relative decrease in rupture was not as low as that observed with KDp rats (Fig. 2D and E). AAA absolute diameter and percentage of aortic diameter increase were also significantly reduced at both week 1 and 2 in EKB rats while only significantly reduced at week 2 in KDt rats (Fig. 4K and Supplementary Fig. S1C). These findings demonstrate that KDt and EKB therapeutic regimens can lead to reduced AAA expansion and risk of rupture, albeit non-significant for EKB.

KDt and EKB rats also demonstrated increased AAA wall media Collagen content (*p* = 0.08 and *p* = 0.02, respectively; Fig. 4L–O), and reduced CCR2 content (*p* = 0.06 and *p* < 0.05, respectively; Fig. 4P–S). No difference was observed in CD68 content across groups (Fig. 4T). Equivalent levels of pro-MMP9 were observed among both treatment groups (Supplementary Fig. S9A and B), but active MMP9 was significantly decreased in KDt and EKB rats (*p* = 0.02 and *p* = 0.001, respectively; Supplementary Figs. S9A and C, S10). Total MMP2 was also notably attenuated in KDt rats (*p* < 0.001), but not in EKB rats (Supplementary Fig. S9A and D). These data suggest that even an abbreviated therapeutic course of ketosis following AAA formation can help stabilize AAAs, preserve aortic wall collagen content, reduce CCR2 tissue content, and promote MMP balance.

### CCR2 is essential for AAA formation and rupture in mice

We used whole body genetic knockdown of *Ccr2* in C57BLK mice, to determine whether CCR2 is essential for AAA formation and rupture. Age-matched male wildtype (*Ccr2*+*/*+) and *Ccr2*−/− adult mice received angiotensin II osmotic pump administration to promote AAA formation^13,33^, and daily BAPN administration to promote AAA rupture^27^ (Supplementary Fig. S11A). Over the subsequent 2 weeks, *Ccr2*−/− mice had significantly reduced incidence of AAA rupture (*p* = 0.003; Supplementary Fig. S11B), and 47% higher survival compared to *Ccr2*+*/*+ (Supplementary Fig. S11C). Moreover, *Ccr2*−/− mice had significantly less AAA formation (*p* < 0.001; Supplementary Fig. S11D). PET/CT with ^64^Cu-DOTA-ECL1i demonstrated significantly reduced CCR2 content in the aorta of *Ccr2*−/− mice (*p* < 0.001; Supplementary Fig. S11E and F). These data confirm that CCR2 is essential for AAA formation and rupture.

## Discussion

**Figure 5:**
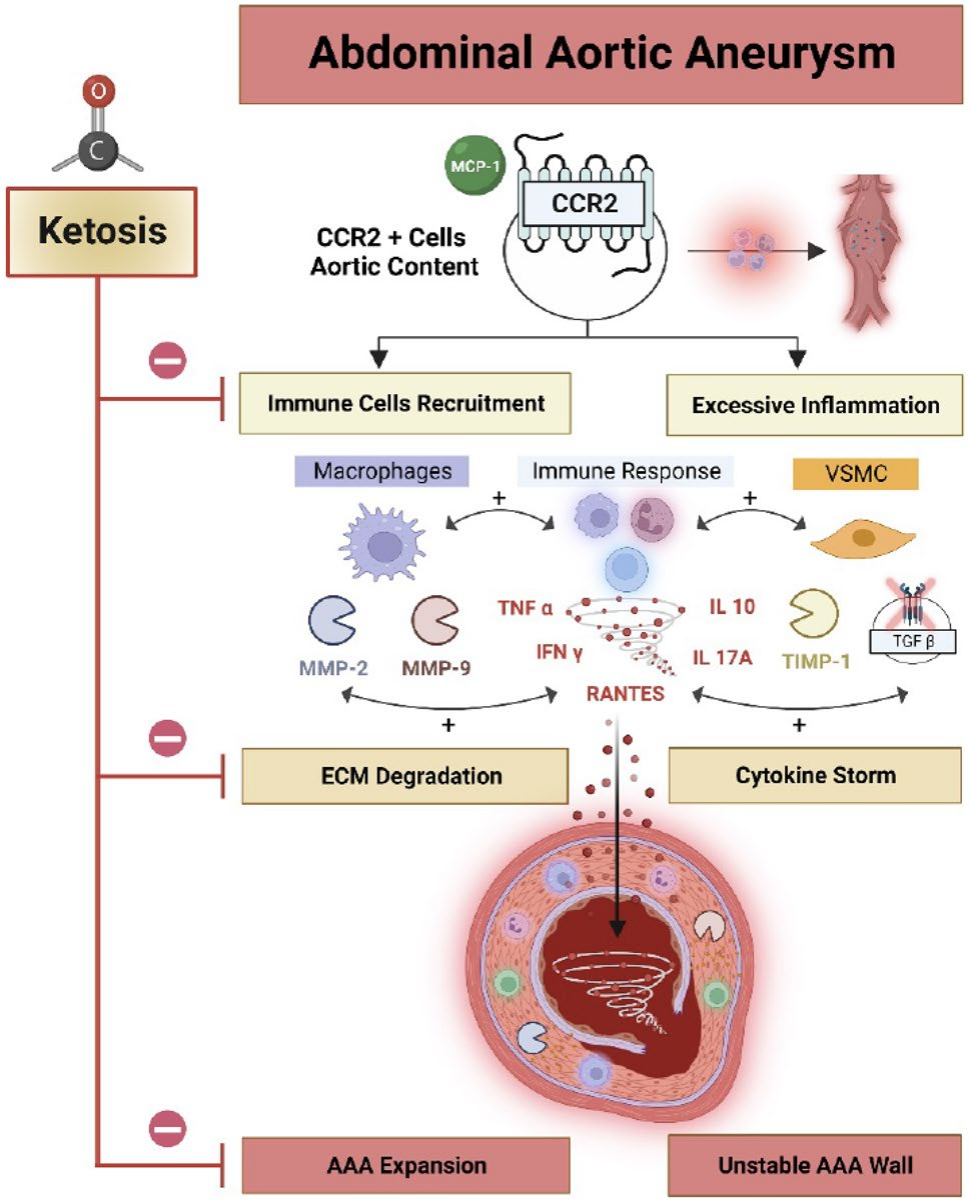
Ketosis impacts AAA expansion and risk of rupture. AAA expansion and risk rupture is influenced by CCR2, which in turn recruits CD68+ pro-inflammatory macrophages, and also leads to cytokine release, and MMP activation. Vascular smooth muscle cells (VSMCs) production of TIMP1 can complex with MMP9 to help balance out the rate of MMP-medicated ECM degradation. Decreased TIMP1/MMP9 complex can lead to higher ECM degradation and AAA expansion. CCR2-mediated release of TNFα, RANTES, IL-10, IL-17A, and IFNγ can further compound AAA tissue stability, and inhibition of TGFβ can progress AAA instability, which further escalates the risk of rupture. Ketosis inhibits inflammation and ECM degradation thereby stabilizes AAA tissue and reduces the risk of rupture. Figure was made using BioRender.com.

Our study demonstrates that *Ccr2* is essential for AAA rupture, and that diet-induced ketosis can reduce CCR2 signaling and decrease AAA pathology progression. Using previously validated, pre-clinical murine mice and rat models for AAA^13,14^, and different ketogenic supplementation strategies, we provide a robust and comprehensive assessment of the impact of dietary ketosis on the AAA inflammatory milieu, aneurysm progression, and the risk of rupture. We also specifically demonstrate that administration of either a ketogenic diet (KDp or KDt) or an oral ketone body supplementation (EKB) can reliably induce systemic ketosis, significantly reduce aortic wall CCR2 and pro-inflammatory cytokines, increase collagen content in the AAA media, and promote an MMP balance that minimizes elastin degradation (Fig. 5).

Rats that received KDp demonstrated the most notable decrease in AAA expansion and risk of rupture. Rats that received KDt and EKB supplements also demonstrated differences in AAA progression, but not to the same extent. There was also mild to moderate variability in the KDt and EKB values of CCR2, CD68, and MMP content in AAA tissue. Administration of BAPN reliably induced AAA rupture and did not appear to confound the impact of ketosis on AAA expansion and the risk of rupture. Additionally, our complementary studies demonstrated that ketosis can impact pro-inflammatory CCR2-mediated signaling mechanisms that can lead to AAA progression. Therefore, this pre-clinical study demonstrates that a low-risk, and relatively easy dietary intervention, can potentially alter the course of AAA disease progression, and provides important insights that can be easily translated to human patients with AAAs who lack an effective medical management strategy.

Ketogenic diets are thought to modify the host systemic energy metabolism to mimic the biochemical impact of starvation. This occurs with the significant increase in serum βHB levels, decrease in blood glucose, and increase in fatty acids^34^. These dietary regimens were originally introduced for the treatment of children with refractory epilepsy, and have now become popular remedies for weight loss, patients with diabetes, obesity, various types of cancer, and among high performance athletes^22,35–38^. Standard ketogenic diets that are devoid of carbohydrates can lead to elevated βHB serum levels that are consistently > 2 mM^38^. Recent studies demonstrate that βHB can serve as an important signaling mediator that can inhibit histone deacetylases^39^, blunt tissue oxidative stress^40,41^, activate G-protein-coupled receptors^42,43^, and regulate various inflammatory mediators^44–47^. In our study we similarly observed that rats with high serum βHB had blunted tissue inflammation and reduced CCR2 content in AAA tissue. This provides compelling evidence that a ketogenic diet may provide stability to AAAs and therefore prevent rupture. Future studies will help determine whether ketogenic diets can similarly stabilize small AAAs in human adults.

Uniquely, our study evaluated the impact of three different ketosis regimens: two types of ketogenic diets (KDp and KDt), and an oral supplement regimen (EKB). KDp included a 1-week priming period prior to AAA formation, that imitates the phenomenon of keto-adaptation that occurs in humans who are maintained longer-term on a ketogenic diet^48^. This regimen aided in determining whether a ketosis primer can have a ‘protective’ impact against AAA formation and expansion. On the other hand, KDt was designed to evaluate the potential ‘therapeutic’ impact of ketosis on expansion and rupture of AAA post-induction with PPE. This regimen would hypothetically be similar to how medical management would be prescribed in humans with small AAAs that do not yet meet the traditional size criteria for operative intervention. In the course of this study, we observed that rats tolerated both KDp and KDt, and that both were successful in inducing a sustained systemic state of ketosis. Interestingly, we observed that both regimens yielded significant reductions in both AAA expansion and the incidence of rupture relative to rats that received SD. However, the longer-term KDp regimen appeared to have a greater protective impact, and a more impressive reduction of CCR2 content in AAA tissue. These findings suggest that the length of diet-induced ketosis may be an important variable in the extent of AAA tissue inflammation reduction and the risk of AAA rupture.

Interestingly, rats that received KDt demonstrated higher levels of serum triglycerides and HDL, whereas rats that received KDp had lipid profiles that were similar to SD. These findings resemble human studies that showed that individuals on shorter regimens of KD tended to have elevated serum lipid profiles, while individuals who were maintained longer term on KD had normalized serum lipid profiles^32^. The variable serum lipid response to KDs has also led to differences in opinion regarding the relative risk assessments of arterial atherosclerosis. There is still an ongoing need for clinical trials to more clearly delineate the risks KD on atheroprogression^32^.

With the recent advent of EKB supplements, oral regimens have been increasingly utilized to manipulate human circulating blood ketone body concentrations for various health benefits^49^. While most studies involving EKB supplementation have traditionally focused on its impact among high-performance athletes^50^, these supplements are increasingly being studied as remedies for conditions such as epilepsy, heart failure, diabetes, and sepsis-related muscle atrophy^51^. Our study evaluated the impact of EKB on AAA expansion and risk of rupture. Interestingly, we observed that EKB not only decreased AAA tissue inflammation, but also reduced AAA expansion and incidence of rupture. The impact of EKB on CCR2 content and AAA rupture differed from the responses observed with KDp and KDt, and we suspect this is because EKB only induced intermittent ketosis (limited to 8 h per day). Similar to prior reports^52^, EKB supplementation did not alter serum lipid profiles. These findings suggest that oral supplementation with ketone bodies can serve as a minimally invasive method for the medical management of AAAs, and is a compelling topic for future human clinical trials—particularly in individuals with a genetic predisposition for impaired lipid metabolism^53–55^.

Our study also suggest that ketosis has a multifaceted impact on aortic wall architecture and function. Inflammation is the major molecular mediator of AAA disease progression (Fig. 5). Previous studies demonstrated that excessive aortic wall inflammation can inhibit reparative signaling, wall fibrosis, and collagen deposition, which in turn can accelerate AAA expansion and lead to a higher risk of rupture^56^. Tissue macrophages are known to promote AAA disease, in particular subsets that highly express CCR2^11^. We as well as others, also previously demonstrated that genetic or molecular targeting of CCR2 can reduce AAA progression^12–14^. Here we provide further completing evidence that CCR2 content indeed correlates with AAA disease progression, and that systemic ketosis in vivo can significantly reduce CCR2 content as well as downstream pro-inflammatory cytokines in AAA tissue.

Previous studies investigating the inflammasome in AAAs demonstrated that TNFα and RANTES are both up-regulated in expanding AAA tissue^57,58^. Inhibition of TNFα appears to decrease aortic wall MMP activity, reduce ECM disruption, and decrease aortic diameter in a murine AAA model^59^. In another study, Empagliflozin, a sodium-glucose cotransporter 2 inhibitor that increases plasma ketone bodies^60,61^, was found to reduce aortic aneurysm diameter and aortic wall RANTES in *Apoe*−/− mice^62^. Similarly, our study observed that diet-induced ketosis can significantly decrease aortic wall pro-inflammatory cytokines TNFα and RANTES, as well as increase aortic wall Collagen content. Although the direct mechanism of action for this is yet to be fully elucidated, we suspect that the molecular interplay between macrophages and other pro-inflammatory cell types contribute to the immune modulation in AAA tissue^63,64^.

A central pathological feature of AAA disease progression is excessive and aberrant extracellular matrix (ECM) remodeling. This results from reduced inactive pro-MMP form, increased MMP activity, and higher rapid ECM breakdown and disruption of the integrity of the aortic wall^65,66^. Previous work demonstrates that MMP2 plays a central role in the formation and early expansion of AAAs, while MMP9 is more related to late AAA expansion and risk of aneurysm rupture^27,67,68^. Synergistic activation of both MMP2 and MMP9 provides an unfavorable environment that can accelerate AAA dilation and lead to a higher risk of aneurysm rupture^69^. Previous studies also demonstrate that ketosis, high serum βHB, and signaling via NF-Kβ, play key roles in suppressing MMP-9 expression in colonic tissue^70^. Our studies build upon this, and demonstrate that along with blunted CCR2 content, ketosis resulted in attenuated active MMP9, reduced total MMP2 content, and increased TIMP1/MMP9 stabilizing complex in AAA tissue (Fig. 5). This is consistent with a recent study that demonstrated CCR2 antagonism similarly downregulated MMP-9 in lung cancer cells, resulting in reduced cellular motility^71^.

Our study also demonstrated a mild-moderate, but non-significant, increase in AAA tissue TGFβ content in rats treated with ketogenic diets (Fig. 5). TGFβ belongs to a superfamily of growth factors that regulate many cellular functions such as cell growth, adhesion, migration, differentiation, and apoptosis^72^. In aortic tissue, TGFβ appears to have a beneficial role. For example, administration of TGFβ neutralizing antibodies appeared to promote excessive monocyte-macrophage infiltration within murine and rat AAA tissue^29,73^, while overexpression or administration of TGFβ1 significantly increased aortic wall collagen deposition^74^, and collagen synthesis in normal arteries^75^.Recent studies demonstrated that TGFβ content is reduced in human AAA tissue^76^, and ketosis promoted TGFβ-induced myocardial fibrosis and Collagen 1 and 3 deposition in spontaneously hypertensive rats^77^. In this context, our study suggests that KDs lead to increased AAA TGFβ content and increase aortic wall Collagen 1 deposition.

We acknowledge that a dietary regimen that mimics a fasting state can be physiologically challenging to evaluate mainly due to its significant impact on energy metabolism. None the less, ad libitum intake of a precisely formulated KD (Supplementary Fig. S12) appears to be the best approach to promote satiety and evaluate the impact of ketogenesis in the most naturally occurring way^78^. For example, similar to prior studies^22,79^, our results demonstrate that an ad libitum ketogenic diet can lead to reduced weight gain. We observed that this reduction mostly occurred during week 1, but by week 2 rats started to gain more weight (Figs. 1C, 2C, and 4C). A similar trend was also observed in sham rats that did not have AAAs (Supplementary Fig. S13). Moreover, we confirmed in all ketogenic diet groups had the hypothesized rise in serum βHB, which is a highly sensitive predictor of ketosis^80^.

Given the ad libitum study design it was nearly impossible to precisely track oral food intake quantities among our different diet groups. We acknowledge that this may have introduced confounding variables into the study, as satiety may have been variably impacted among the diet intervention groups. Nevertheless, we observed that rats that received either KD or EKB had no difference in total cholesterol or LDL (Fig. 4E and F). On the other hand, in addition to elevated serum βHB, rats that received KD had elevated serum FFAs, which is typically released from adipose tissue to serve as an alternative fuel and is converted into acetoacetate, βHB, and acetone^81,82^. These observations provide supporting evidence that KD had its intended effect, despite the potential limitations of an ad libitum study design.

Furthermore, we acknowledge that all our data was derived from male pre-clinical rodent models that may not be necessarily representative of human AAA pathophysiology. Our male rat AAA rupture model was previously validated and shown to be the most reliable and consistent AAA rupture model to date. Given the known impact of estrogen on AAAs^1,3^, there is currently no reliable AAA rupture model in female rodents and is an area that requires further investigation and development. In the future, we hope to conduct similar studies using female rodents to determine the impact of sex on our study findings. Additionally, our studies did not systematically evaluate arterial blood pressure. This would have required more sophisticated monitoring apparatuses, and possibly indwelling sensors that evaluate continuous telemetry. While such monitoring systems are feasible for shorter experimental protocols, our 2–3 week experimental protocol would have greatly complicated the experimental design and led to several confounding variables. Instead, we serially monitored AAA endpoints via ultrasound, which provided reliable and reproducible data. Lastly, our study used a single composition for the ketogenic diet intervention. We acknowledge that this is not fully representative of the variety of human lipid and oil-based ketogenic diets, but this was selected to maintain consistency and adherence within all rodent study groups.

In conclusion, this study demonstrates that KDs and EKB supplementation can significantly reduce AAA expansion and reduce the incidence of AAA rupture. Importantly, a ketogenic priming period appears to be further protective, while EKB appears to be less effective than other dietary regimens. KDs reduced CCR2 content, promoted MMP balance, and attenuated ECM degradation in AAA tissue. These findings provide the impetus for future pre-clinical and clinical studies geared to determine the role of ketosis as a medical management tool for human patients with AAAs that do not yet meet the criteria for surgical intervention.

## Methods

### Animals

Male Sprague–Dawley rats (200–300 g) were obtained from Charles River Laboratories (Wilmington, MA; see supplemental methods). Adult 12-week-old, male, *Ccr2*−/− and *Ccr2*+*/*+ mice on a C57BL/6 background were obtained from The Jackson Laboratory (Bar Harbor, ME; see supplementary methods). All animals were housed at 21 °C in a 12/12 h light/dark cycle and had access to food and water ad libitum. Anesthesia was administered with a mixture of ~ 1.5% isoflurane and oxygen for all procedures. The core body temperature was continuously monitored and maintained with a heating pad at 37 °C. Use of all animal experiments were performed in accordance with relevant guidelines and regulations, were approved by the Institutional Animal Care and Use Committee (IACUC) at Washington University School of Medicine in St. Louis and reported in accordance with ARRIVE guidelines. At the conclusion of studies, live animals were sacrificed appropriately using anesthetic agents and cervical dislocation.

### Induction of AAA and sham-control models

AAA rats were induced to develop infrarenal AAAs via an established model using porcine pancreatic elastase (PPE; 12 U/mL), while sham-control rats (n = 8) were exposed to heat-inactivated PPE (12 U/mL, heated at 90 °C for 45 min; Supplementary Fig. S13) as previously described^28^. Ventral abdominal wall laparotomy is performed, and the infrarenal abdominal aorta was exposed (Supplementary Fig. S14). A customized polyethylene catheter (Braintree Scientific, Braintree, MA) was introduced through an infrarenal aortotomy, and elastase was infused into the isolated aortic segment for 30 min. The exposed aortic segment was dilated to a maximal diameter, and constant pressure was maintained with the use of a syringe pump. Using a video micrometer, the baseline maximum aortic diameter was measured. After 14 days, all rat aortas were re-exposed via ventral abdominal laparotomy, maximal aortic diameters were measured, and aortic tissue was harvested for further analysis (Supplementary Fig. S14).

### Promoting AAA rupture model

As previously described, BAPN is reported to promote AAA tissue inflammation by day 6, and AAA rupture between days 7 and 14, but unlikely to cause rupture after day 14 if it has not already occurred^14^. We therefore promoted AAA rupture with daily administration of BAPN on a specific cohort of rats (RAAA) starting 3 days before PPE exposure. Through drinking water, 300 mg BAPN was administered daily (0.3% BAPN in 25 mL water consumed per day by a 250 g rat)^83^. AAA growth was monitored for 1 week (6–7 days) or 2 weeks (14 days). At the 1 or 2-week timepoints, rats were sacrificed, AAA diameters were evaluated, and whole blood and aortic tissue were collected for further analysis. Rats that developed ruptured AAAs (RAAA) during the study period promptly underwent necropsy to confirm and analyze the pathology (Supplementary Fig. S14). By day 14, rats that survived (non-ruptured AAA; NRAAA) were evaluated via open surgical laparotomy. Tissue harvested at week 1, prior to rupture, was mainly used to assess AAA tissue inflammation (Supplementary Fig. S2).

### Animal diets

We evaluated four different dietary interventions. First, control groups in the AAA (n = 5) and RAAA (n = 12) cohorts were fed with a standard chow diet (SD). Second, experimental groups in the AAA (n = 6) and RAAA (n = 8) cohorts were fed a very high fat diet with almost no carbohydrate, also known as a classic ketogenic diet one week prior to PPE exposure to induce a ‘priming’ keto-adapted status^84^, before AAA induction (Supplementary Fig. S12). Ketogenic diet was then maintained prospectively in these groups following AAA formation. Third, experimental groups in the RAAA (n = 9) cohorts were separately started on ketogenic diets as a ‘treatment’ intervention 3 days after AAA induction. Lastly, experimental RAAA rats were administered a SD along with exogenous ketone body (EKB) supplementation starting 3 days after PPE exposure: RAAA + EKB (n = 10). As previously described^52^, this EKB supplementation was performed with daily intragastric gavage of 1,3-Butanediol^85^ (BD; 5 g per kg dose; Prod # B84785-100ML; Sigma-Aldrich, St. Louis, MO) and rats achieved a ketosis state only for 8 h per day (Supplementary Fig. S12).

### Synthesis and radiolabeling of DOTA-ECL1i

The ECL1i peptide (LGTFLKC) was synthesized from D-form amino acids by CPC Scientific (Sunnyvale, CA). DOTA-ECL1i was prepared following our previous report. Copper-64 (64Cu, t1/2 = 12.7 h) was produced by the Washington University Cyclotron Facility. The DOTA-ECL1i conjugate was radiolabeled with 64CuCl2 (64Cu-DOTA-ECL1) as described, and the radiochemical purity was determined by radio-HPLC^14^.

### Animal PET/CT imaging analysis

Dynamic PET scan and corresponding CT images were obtained using Inveon MM PET/CT (Siemens, Malvern, PA) at 45–60 min after a tail vein injection of 64Cu-DOTA-ECL1i (selective CCR2-targeting radiotracer; 11.1 MBq per rat) to minimize the effect of blood retention on AAA uptake. To localize tracer uptake, a CT contrast agent (1.0 mL, eXIA 160XL, Binitio, Canada) was administrated via tail vein after PET imaging. Contrast-enhanced CT (Bin of 2, 90 mm axial FOV, 60 kV, 500 μA, 500 ms exposure time, 10 ms settle time, no magnification, pixel size: 80–100 μm) was performed. The AAA uptake was calculated as standardized uptake value (SUV) in 3-dimensional regions of interest from PET images without correction for partial volume effect using Inveon Research Workplace software (Siemens). Imaging specificity of CCR2 tracer binding detection within the AAA wall was previously identified by CT contrast agent using the same rat AAA model^14^. Dynamic (0–90 min) 18F-fluorodeoxyglucose (41.1 MBq per rat) PET was also performed in AAA rats at week 1 and 2 post-PPE exposure to identify areas with high glucose metabolism. Only a specific number of each group of rats received PET/CT imaging.

### Ultrasound aortic assessments

Noninvasive ultrasound (GE, 12 MHz Zonare, Mountain View, CA), was used to evaluate serial aortic maximum diameter measurements. Relative to baseline aortic diameter prior to PPE exposure, the percentage increase in aortic diameter was evaluated at 1- and 2-weeks post-PPE exposure. As previously described, aortic aneurysms were defined as > 100% increase in the aortic maximum diameter relative to baseline diameter^28,86^.

### Blood βHB assessments

Animal state of ketosis was evaluated via whole blood D-βHB (Keto-MoJo blood ketone meter; Keto-Mojo, Napa, CA, USA) concentrations^79^. Tail vein puncture was used for blood sample, which was tested on day 0 pre-PPE exposure, and then 1- and 2-weeks following AAA induction. βHB values > 0.5 mmol/L were indicative of ketosis.

### Serum free fatty acids and lipids measurements

Whole blood samples (1 mL) were collected from Inferior Vena Cava (IVC) puncture at 2-week timepoint (14 days), and centrifugated for serum isolation. Serum samples were sent to Washington University Core Lab for analysis of free fatty acids (FFA), total cholesterol (TC), direct low-density lipoprotein (LDL), triglycerides (TG) and high-density lipoprotein (HDL).

### Animal weight

Animal whole body weights were evaluated at day 0 pre-PPE exposure, and 1 and 2 weeks followed AAA induction. Body weight was evaluated by the difference between the values at the baseline (Day 0) and the values at week 1 and 2 respectively and then divided by the baseline to assess difference. All these absolute numbers were then multiplied by 100 to present it as the percentage of difference in weight throughout the time of the study.

### Histology and immunostaining

Aortic tissue was harvested from all animals. AAA tissue was fixed in Histochoice (VWR), and paraffin embedded. Paraffin blocks were sectioned at 5 μm, and deparaffinized. Processing for antigen retrieval was performed with Sodium Citrate solution, pH 6.0, for 10 min. Tissue sections were blocked with 10% serum, and sections were incubated with primary antibody anti-CD68, 1:100 (Bio-Rad, MCA341GA), CCR2, 1:200 (Novus-Bio, NBP1-48338), CD86 (Thermo-Fisher, #942-MSM-P0) and CD206 (Thermo-Fisher, #187004-1-AP). For Immunofluorescence, sections were incubated with donkey anti mouse (Alexa Fluor 647), and donkey anti rabbit (Cy3) (Jackson ImmunoResearch Laboratories). All sections were imaged using a Leica THUNDER Imager 3D tissue microscope system. Sections were then incubated with anti-mouse secondary antibodies conjugated with HRP (Cell Signaling), DAB peroxidase substrate kit (Vector Laboratories), and counter stained with hematoxylin, imaged using an Olympus fluorescent microscope system. To evaluate AAA tissue morphology and pathology, tissue sections were also evaluated using Hematoxylin and Eosin (H&E), Mason Trichrome (MT) and Verhoeff-Van Gieson (VVG), imaged using NanoZoomer, Supported by the Alafi Neuroimaging Laboratory. All AAA wall staining intensity was analyzed in 3 random ROIs, quantified with Image J, and normalized to the total area of the aortic wall media.

### ELISA and cytokine array assessments

AAA tissue protein was extracted using RIPA buffer with proteinase inhibitor (Sigma #MCL1). Protein quantification was done by Bradford assay. For each AAA tissue samples 25ug of protein was analyzed for Tissue Inhibitor of Metalloproteinases 1 (TIMP1)-specific ELISA (RayBiotech, ELR-TIMP1-CL-2), MMP-9 (MyBioSource, MBS722532), CD68 (MyBioSource, MBS705029) and Cytokine multiplex assay (Millipore, RECYTMAG-65 K) using manufacturer instructions.

### MMP activity zymography

For each AAA tissue sample, 25ug of protein was loaded on wells of 10% Gelatin Zymogram electrophoresis gels. Gels were then incubated in Zymogram renature buffer for 30 min, followed by 36 h of Zymogram development buffer at 37 °C. Gels were then stained with Coomassie Brilliant Blue R-25 solution from BioRad for 30 min, followed by de-staining buffer (20% Methanol, 20% Acetic acid, 60% DI water) until MMP bands were visualized. Gels were scanned on BioRad Chemi doc and analyzed using ImageJ software.

### Immunoblotting

From each AAA sample 25 μg of denatured protein was separated on 4–12% polyacrylamide by electrophoresis and then transferred to PVDF membrane. The membranes were then incubated with collagen type-1 (Millipore #ABT123, 1:2000), TGF-β1 (Sigma #AV44268, 1:1000), α-SMA (Sigma #A2547, 1:1000) and KFkB (Thermo-Fisher #PA516545). GAPDH (Sigma # G9545, 0.1 mg/ml) and Caveolin-1 (Santa Cruz # sc-53564, 1:1000) were used as loading controls. Membranes were treated with HRP-conjugated secondary antibody at room temperature for 1 h and evaluated with chemiluminescence. Blot band intensities were analyzed using ImageJ software.

### Statistics

All data are presented as the mean ± SD. Most group comparisons were performed using unpaired t test. For comparisons that included one endpoint in more than one animal/diet groups, an ordinary one-way ANOVA with multiple comparison was performed. For comparisons that included more than one endpoint in more than one animal/diet group, we utilized a two-way ANOVA with multiple comparison. Data was considered statistically significant with *p* ≤ 0.05. Kaplan–Meier curve was generated to assess the survival of BAPN-exposed animals. GraphPad Prism 9 (La Jolla, CA) was used for all statistical analyses and graphical data representations. MT and VVG cross section staining’s were analyzed using ImageJ software by color deconvolution, adjust threshold and region of interest assessment of the AAA to analyze collagen deposition and elastin degradation. Immunofluorescence in AAA wall tissue-stained with CD-68 (red channel), CCR2 (green channel), and DAPI (blue channel) were also analyzed by ImageJ software using split channels, inversion and threshold adjustment and shown as mean intensity (content) for each staining in the specific regions of interest (ROI).

### Supplementary Information

Supplementary Information.

## Data Availability

The datasets generated and/or analyzed during the current study are available from the corresponding author on reasonable request.
