# Supplementary material for: Ketosis prevents abdominal aortic aneurysm rupture through C–C chemokine receptor type 2 downregulation and enhanced extracellular matrix balance
Source: Sci Rep. 2024 Jan 16;14:1438. doi: 10.1038/s41598-024-51996-7 (PMC10791699; doi:10.1038/s41598-024-51996-7)
Supplement: Supplementary file 1 — Supplementary Information. [file 41598_2024_51996_MOESM1_ESM.docx]

**Supplementary Information for**

**Ketosis Prevents Abdominal Aortic Aneurysm Rupture Through C-C Chemokine Receptor Type 2 Downregulation and Enhanced Extracellular Matrix Balance**

**Sergio Sastriques-Dunlop^1*^, Santiago Elizondo-Benedetto^1*^, Batool Arif^1^, Rodrigo Meade^1^, Mohamed S. Zaghloul^1^, Hannah Luehmann^2^, Gyu S. Heo^2^, Sean J. English^1^, Yongjian Liu^2^, Mohamed A. Zayed^1,2,3,4,5^**

Corresponding Author Email: zayedm@wustl.edu

**This PDF file includes:**

Supplemental Results

Supplemental Methods

Supplementary Figures S1-14

**SUPPLEMENTARY INFORMATION**

**Supplemental Results**

**Impact of Ketogenic Diet on AAA Absolute Diameter**

At week 1, both KDp and SD rats that were not provided with BAPN, demonstrated similar AAA absolute diameters (mm). However, at week 2, KDp rats had significantly lower AAA diameters (p = 0.008; **Supplementary Fig. S1A**). Moreover, a different cohort of KDp rats that received BAPN for rupture stimulation also demonstrated significantly decreased AAA absolute diameters when compared to SD rats that received BAPN (**Supplementary Fig. S1B**). Similarly, AAA absolute diameter was decreased at week 1 and 2 in both KDt and EKB rats when compared to SD rats, and differences were more profound in week 2 (**Supplementary Fig. S1C**).

**Impact of a KDp and EKB on AAA Diameter and ECM Staining at Week 1**

At week 1 it is anticipated that AAA tissue will have maximal aortic wall inflammation (28). We therefore evaluated PPE and BAPN rats at this time point to evaluate differences in AAA diameter and collagen deposition. Rats maintained on SD (N=10), KDp (N=8), and EKB (N=7) were harvested at week 1 post-PPE and daily BAPN treatment (**Supplementary Fig. S2A**). Rats maintained on KDp were consistently in a state of ketosis during days 0-6, but rats maintained on EKA remained in ketosis just for 8-hour per day (**Supplementary Fig. S2B**). KDp and EKB rats harvested at week 1 had significantly reduced weights (p < 0.001; **Supplementary Fig. S2C**). Aneurysm diameter at week 1 was significantly decreased in both KDp and EKB rats compared to SD rats (p = 0.004 and p < 0.001 respectively; **Supplementary Fig. S2D)**. However, aortic wall media demonstrated equivalent amounts of MT-stained collagen between all diet groups (**Supplementary Fig. S2E**).

**Impact of BAPN on Ketogenic Diet Consumption**

We did not observe that BAPN had a significant impact on body weight gain and blood ketosis levels. At week 0, 1 and 2, rats fed a SD that were not exposed to BAPN (SD No BAPN) demonstrated similar levels of blood βHB when compared to SD rats that received BAPN (SD BAPN). Similarly, KDp rats that were not exposed to BAPN (KDp No BAPN) had comparable values of blood βHB to those KDp that received BAPN (KDp BAPN; **Supplementary Fig. S3A**). Therefore, KDp No BAPN and KDp BAPN groups were in ketosis for the same study duration (**Supplementary Fig. S3A**). Body weight gain at week 0, 1 and 2, was also unchanged between SD and KDp rats that were or were not receiving BAPN (**Supplementary Fig. S3B**). Therefore, it does not appear that BAPN was a significant confounder of KD or SD consumption during the course of the study.

**F- fluorodeoxyglucose PET/CT assessment and uptake quantification**

^18^ F-fluorodeoxyglucose (FDG) is a non-specific tracer for areas of glucose metabolism we have previously used to compare with our targeted tracer. FDG PET/CT was performed in KDp and SD rats at week 1, and revealed comparable uptake (p = ns) to that of ^64^Cu-DOTA-ECL1i, which was also consistent with our previous reports in the same rat AAA model (**Supplementary Fig. S4A & B**).

**Ketogenic diet on CD68 + Cells: no correlation to MMP9 levels in rats AAA wall analysis**

Rats that received a ketogenic diet demonstrated less CD68+ cells via quantitative and qualitative analysis (**Supplementary Fig. S5**). Although an increased volume of CD68+ cells in the AAA wall and high levels of MMP9 appeared to be predictors of severe progression of the aneurysm, we performed a correlation analysis and did not found a good correrlation between these crucial markers (**Supplementary Fig. S6**). This can be explained not only by the highly variability observed in AAA disease but to the fact that macrophages are not the only cells in AAA that produces MMP9 (68-69), therefore other cells may also be increased in the aneurysm wall and could be causing this events.

**Ketogenic diet on macrophage phenotype (CD68 and CD206+ cells)**

AAA tissue specimens with **CD86 marker** (pro-inflammatory or M1 macrophages) and **CD206** marker (pro-regenerative anti-inflammatory or M2 macrophages) were assessed.

Interestingly, this analysis demonstrated a significantly reduced infiltration of CD86+ macrophages population (pro-inflammatory or M1 phenotype) as well as a modest increase in CD206+ macrophages (pro-regenerative anti-inflammatory or M2) within the AAA wall of KDp rats (p = 0.04 and p = ns respectively; **Supplementary Fig. 7A-D**) suggesting that KD modulated CCR2 and promoted a pro-regenerative anti-inflammatory (M2) mileu and decreased the pro-inflammatory macrophage infiltration to further decreased AAA inflammation, MMP secretion and eventual rupture.

**Impact of EKB on MMPs and Inflammation at Week 1**

Gelatin zymography was performed on AAA tissue from rats treated with daily BAPN and maintained on EKB for 1 week. Pro-MMP9 as well as total-MMP9 levels where equivalent between SD and EKB rats (**Supplementary Fig. S11A & D**). However, active MMP9 was significantly reduced in EKB rats (p = 0.01; **Supplementary Fig. S11B & D**). Similarly, total MMP 2 was also reduced in EKB rats (p < 0.001; **Supplementary Fig. S11C & D**). AAA tissue ELISA demonstrated significantly decreased TIMP1 content in EKB rats (p = 0.01; **Supplementary Fig. S11E**). These findings demonstrate that exogenous ketosis can decrease MMP9 activation. Additionally, AAA tissue from rats maintained on EKB demonstrated significantly decreased TNFα (p = 0.001; **Supplementary Fig. S11F**), MCP-1(p = 0.02; **Supplementary Fig. S11G**), RANTES (p < 0.001; **Supplementary Fig. S11H**), and cytokine IL-10 (p < 0.001; **Supplementary Fig. S11I**).

**Ketosis effect on Sham (Heat-Inactivated Elastase) model in rats**

To evaluate the impact of a ketogenic diet in non-AAA induced rats, we compared a SD to rats maintained on a ketogenic diet prior to HIE exposure (KDp; Supplementary Fig. S12A). KDp achieved a state of sustained ketosis from week -1 until week 2 (Supplementary Fig. S12B) and caused no significant modifications in weight gain throughout studied timepoints (Supplementary Fig. S12C). By week 2, no AAA development was found in both groups (Supplementary Fig. S12D). Serum FFA measurement showed a significantly increased levels in the KDp group (p < 0.001; Supplementary Fig. S12E) , whereas the lipid profile, such as total cholesterol, LDL, triglycerides and HDL remained unchanged (Supplementary Fig. S12F-I). This result suggest that FFA levels highly correlated to ketogenesis and that after 3 weeks on a KD, this diet appears to have not detrimental effect on lipid profile. Moreover, ELISA cytokine array demonstrated a modest decrease in IL-1 β, IL-10 and MCP-1 (p = ns; Supplementary Fig. S12J-L respectively), rest cytokines evaluated were found unchanged (Supplementary Fig. S12M&N). Total MMP9 and MMP2 were also unchanged (Supplementary Fig. S12O-Q). Interestingly, Collagen I protein content was found to be significantly decreased in KDp rats (p = 0.04; Supplementary Fig. S12R), whereas α-SMA and TGF-β remain unchanged (Supplementary Fig. S12S-U). This model serves as a control for the observed impact of a ketogenic diet in AAA disease.

**Supplemental Methods**

**Modified AAA Surgical Model Formation in Rats**

On Day 0, utilizing a midline abdominal incision, the rat infrarenal aorta is exposed from the left renal vein to the aortic bifurcations. The left iliolumbar artery and vein are ligated with 7-0 prolene sutures and divided. Other lateral and lumbar aortic branches are also ligated with 7-0 prolene suture. To isolate the abdominal aorta, three separate pieces of 3-0 silk sutures are placed around the aorta just below the left renal, mid-abdominal aorta, and just above the aortic bifurcation as depicted in **Supplementary Fig. 13A**. After temporarily tying down the proximal and distal suture sites a ventral aortotomy is performed with a 30G x 1/2” needle puncture (Becton Dickinson, Franklin Lakes, NJ) in the mid-abdominal aorta and blood is drained from the isolated segment of aorta. A custom polyethylene catheter (Braintree Scientific, Braintree, MA) is then introduced through the aortotomy, and after securing the tube in place with the mid-aortic 3-0 silk suture, PPE (12U/mL) is instilled into the isolated aortic segment for 30 minutes. The isolated segment is dilated to a diameter approximately 50% greater than baseline, and constant pressure is maintained with the use of a syringe pump (**Supplementary Fig. 13A**). Using a microscopic measurement tool, the aortic diameter is measured just distal to the crossing left renal vein, just proximal to the aortic bifurcation, and also in the mid-abdominal aorta. At the end of the procedure, using standard microsurgical techniques, the aortotomy was closed with an interrupted 10-0 nylon suture. The proximal and distal aortic 3-0 silk sutures are then released, and blood flow is reestablished in the abdominal aorta. Postoperatively, rats are given sterile warmed fluids, 1−2 mL, IP, to replace the intraoperative minimal blood loss and to remoisten the abdominal cavity. The ventral abdomen is then closed in a continuous fashion in two layers, with muscle and fascia closed with a 4-0 vicryl suture, and skin closed with a 4-0 nylon monofilament suture.

**Mice AAA rupture model and assessment**

Adult 12-week-old, male, *Ccr2-/-* and *Ccr2+/+* (wildtype) mice on a C57BL/6 background were obtained from The Jackson Laboratory. AAA formation was induced via an established model using porcine pancreatic elastase (PPE; 12 U/mL) as described above in rat AAA surgery. Heat inactivated PPE was used for sham *Ccr2+/+* mice. Additionally, a subcutaneous osmotic pumps was implanted (Alzet 1004, Durect Corp, Cupertino, CA) in all mice to elute angiotensin II (Sigma Aldrich Inc, St. Louis, MO) at 2000 ng/kg/min for 14 days as previously described^14^. All mice included in this study also received 0.3% BAPN dissolved in drinking water, which was initiated 3 days prior to osmotic pump placement and continued throughout the experimental course (**Supplementary Fig. S10A**). Using a video micrometer, the baseline maximum aortic diameter was measured. After 14 days, all mouse aortas were re-exposed via ventral abdominal laparotomy, maximal aortic diameters was measured, and aortic tissue was harvested for further analysis (**Supplementary Fig. S10A**). PET/CT scan was also performed to qualitatively and quantiatively evaluate CCR2 content within the AAA wall.

**Postoperative Analgesia & Euthanasia**

Rats were treated with pre-operative analgesia (Buprenorphine SR) 1 hour prior to the AAA induction procedure. Buprenorphine SR provides 72 hours of pain relief and was dosed at 1 mg/kg and administered subcutaneously. At the time of euthanasia, animals were anesthetized with isoflurane, and the ventral abdominal incision was reopened. The AAA was dissected free from the surrounding tissue, and blood was collected from the inferior vena cava using a 25-gauge needle and 1 mL syringe (Becton-Dickinson, Franklin Lakes, NJ). The aorta was then excised from the level of the left renal vein to the aortic bifurcation for tissue processing and analysis. Suprarenal and thoracic Aorta were also collected and processed accordingly for some treatment groups.

**Animal Necroscopy**

Animals that ruptured over the course of the study were promptly assessed and defined as ruptured AAA animals. A left retroperitoneal hematoma was indicative of a ruptured AAA (**Supplemental Figure 13B & C**).

**Non-invasive Ultrasound Aortic Diameter Assessments**

The intraluminal diameter of the aorta immediately distal to the crossing left renal vein, immediately proximal to the aortic bifurcation, and midway between these two locations was measured with ultrasound (12MHz Zonare, Mountain View, CA) as previously described (80). The percent increase in aortic diameter was determined considering the average baseline intraluminal aortic diameter and the maximum aortic diameter at week 1 and week 2 post-PPE exposure.

**Animal Diet Composition**

The standard chow diet (SD) utilized in this study was purchased from PicoLab® (PicoLab Rodent Diet 20 #5053) and provided by the Division of Comparative Medicine (DCM) in pellet form. The number of calories provided by this diet was 24.5% from protein, 13.1% from fat and 62.4% from carbohydrates (**Supplementary Fig. S14A & B**). The ketogenic diet (KD) utilized in this study was purchased from ENVIGO (ENVIGO Ketogenic Diet TD. #96355) and administered by our Laboratory in a powder (paste) form through specific rodent food containers. The number of calories provided by this diet are 9.2% from protein, 90.5% from fat and 0.3% from carbohydrates. These diets were provided ad libitum. Apart from the difference observed in weight loss, the ketogenic diet produced a lighter color in the stools, while the SD stools were dark black (**Supplementary Fig. S14C & D**). EKB, 1,3-Butanediol, (BD; 5g per kg dose; Prod # B84785-100ML, St. Louis, MO) was provided by daily oral gavage every day starting three days post-PPE exposure until the end of the study. Mechanistically, BD is a racemic βHB precursor; widely available, nontoxic di-alcohol and component part of the R, S-1,3-butanediol acetoacetate diester. Following ingestion, BD is passively absorbed in the gut and subsequently increases blood R- and S-BD, before rapidly undergoing hepatic conversion to the isotopic enantiomers, D-βHB and L-βHB. By this manner, BD increases blood βHB concentration to ~1.0 mmol/L within 30 minutes in experimental animals as well as human subjects^83,84^. In our experimental rats, one dose per day of BD elevated ketone bodies over 0.5mmol/L for 8 hours per day (**Supplementary Fig. S14E & F**).

**
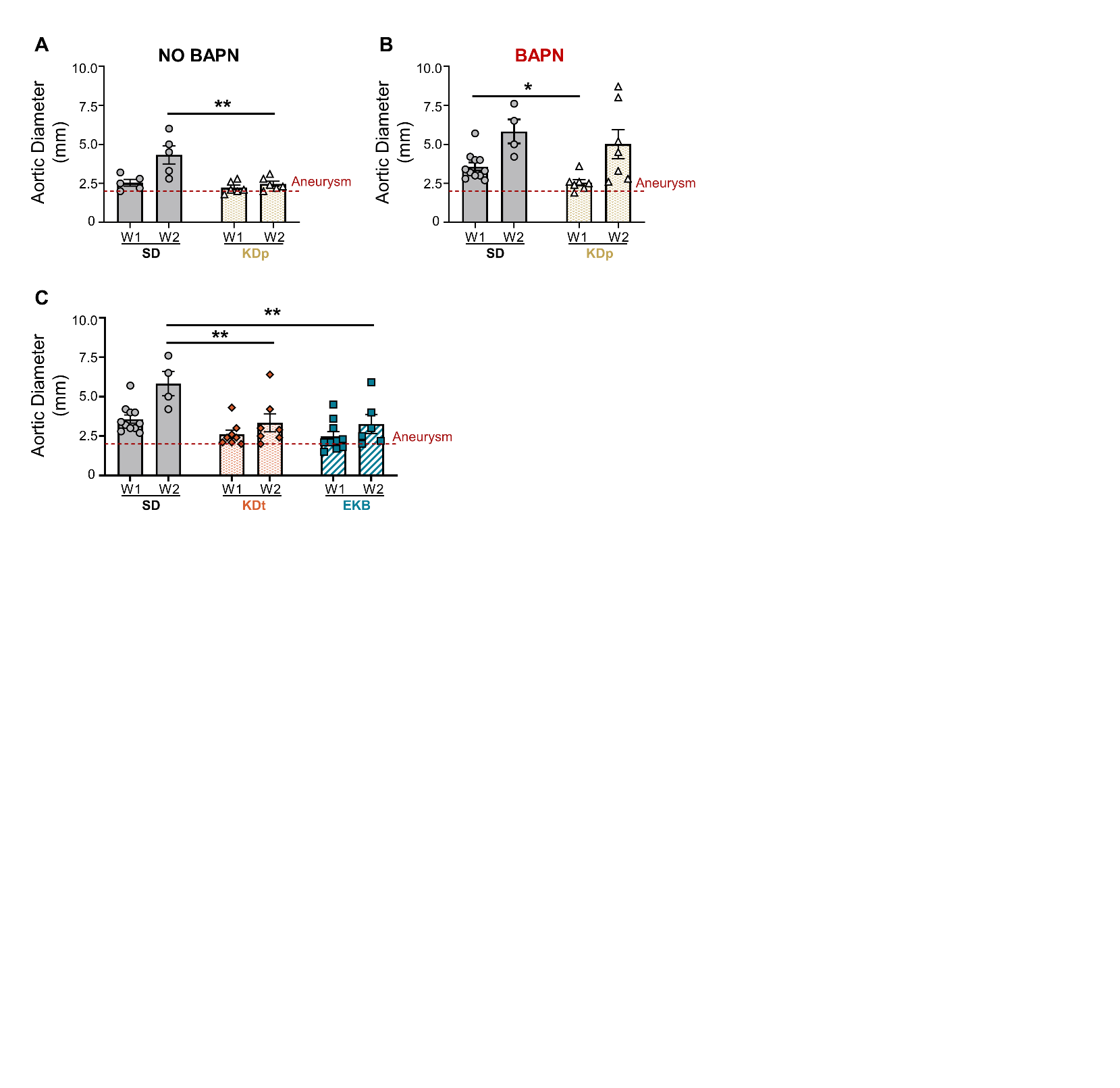
**

**Supplementary Figure S1: Differences in AAA absolute diameter between groups. (A)** AAA absolute diameter (mm) for rats that did not receive BAPN, at week 1: SD (2.54 ± 0.5mm) vs KDp (2.23 ± 0.4mm; p=ns), and at week 2: SD (4.32 ± 1.3mm) vs KDp (2.5 ± 0.4 mm; p=0.008). (**B**) AAA absolute diameter for rats that received BAPN for rupture stimulation, at week 1: SD (3.6 ± 0.9mm) vs KDp (2.54 ± 0.5mm; p=0.01), and at week 2: SD (5.82 ± 1.5mm) vs KDp (5.04 ± 2.5mm; p=ns). (**C**) AAA absolute diameter for rats that received BAPN, at week 1: SD vs KDt vs EKB (3.6 ± 0.9mm vs 2.61 ± 0.7mm vs 2.48 ± 0.9mm; p=ns) and at week 2 (5.82 ± 1.5mm vs 3.34 ± 1.5mm vs 3.26 ± 1.5mm; p=0.003). Data presented as mean ± standard deviation. ns>0.05, *p<0.05, **p<0.01, ***p<0.001 using students t test, ordinary one-way ANOVA, or two-way ANOVA with multiple comparison.


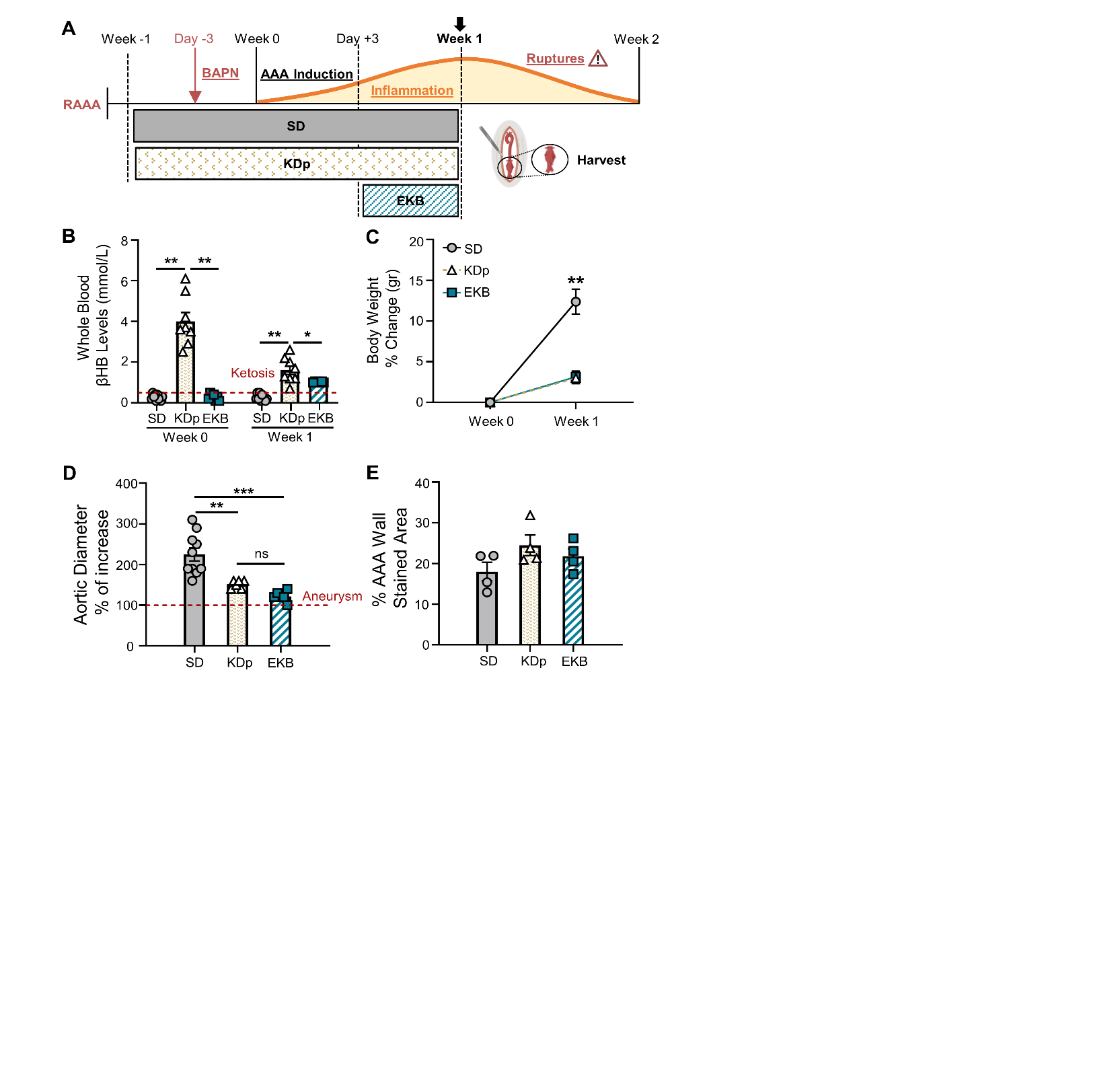


**Supplementary Figure S2: Impact of a KDp and EKB on AAA Expansion at Week 1.** **(A)** Rats underwent exposure to PPE for AAA induction and BAPN was administered daily. Rats also received a priming ketogenic diet (KDp) or exogenous supplement (EKB), and aortic tissue was harvested at week 1 before aneurysm rupture could occur and at the anticipated time of aortic wall peak inflammation. (**B**) Ketosis (βHB whole blood levels > 0.5 mM/L) was verified at week 0 in SD (0.3±0.1) vs KDp (4±1.2; p<0.01) and EKB rats (0.3±0.1; p=ns) and at week 1 in SD (0.3±0.1) vs KDp (1.6±0.6; p<0.01) and EKB (1±0.02; p<0.05). (**C**) Body weight difference in SD (12.3±4.8) vs KDp (3±1.9; p<0.001) and EKB rats (3.1±1.9; p<0.001) at week 1. (**D**) Percent aortic diameter in SD (225±51) vs KDp (152±10; p=0.002) and EKB rats (122±13; p<0,001) at week 1 before harvesting. (**E**) Trichrome staining quantification of AAA tissue in rats fed SD (18±4.5) vs KDp (24±5; p=ns) and EKB (22±4; p=ns). Data presented as mean ± standard deviation. ns > 0.05, *p < 0.05, **p < 0.01, ***p < 0.001 using ordinary one-way ANOVA or two-way ANOVA with multiple comparisons.

**
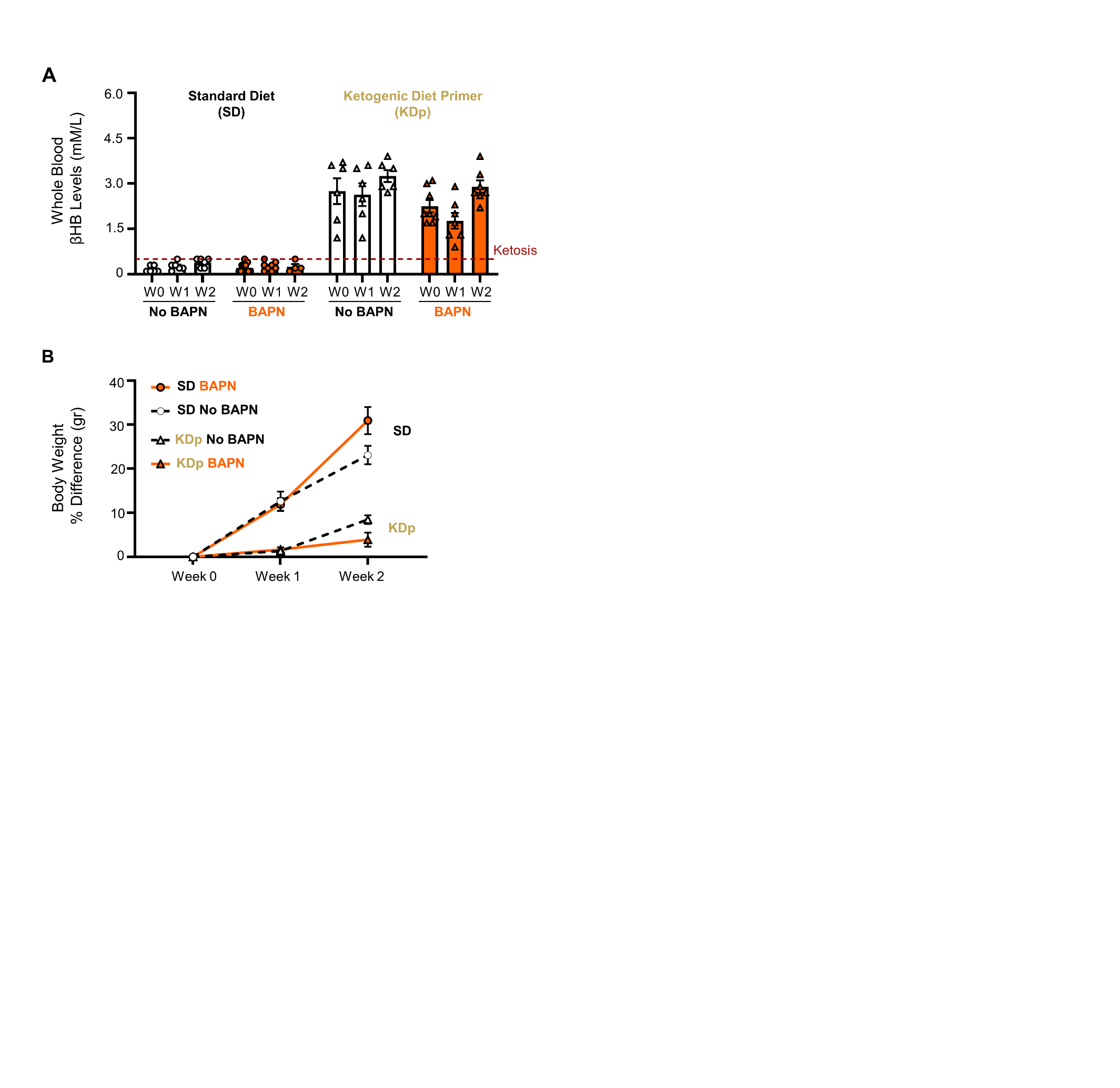
**

**Supplementary Figure S3: Ketosis and body weight gain values are independent of BAPN treatment.** (**A**) Blood βHB levels were evaluated at week 0, 1 and 2 in SD No BAPN (0.2±0.1, 0.3±0.1 and 0.4±0.1) and SD BAPN (0.2*±*0.1, 0.3±0.1 and 0.2±0.1; p=ns). KDp No BAPN rats (3±1, 3±1 and 3±0.5) vs KDp BAPN rats (2±0.5, 1.8±0.7 and 3±0.5; p = ns). (**B**) Percent body weight difference at week 1 and 2 in SD No BAPN (13±5 and 23±5) vs SD BAPN rats (12±5 and 31±6; p=ns). KDp No BAPN (2±1.3 and 8±2) vs KDp BAPN (2±1.3 and 4±3; p=ns). Data presented as mean ± standard deviation. ns>0.05, *p<0.05, **p<0.01, ***p<0.001 using Students t test, ordinary one-way ANOVA, or two-way ANOVA with multiple comparisons.


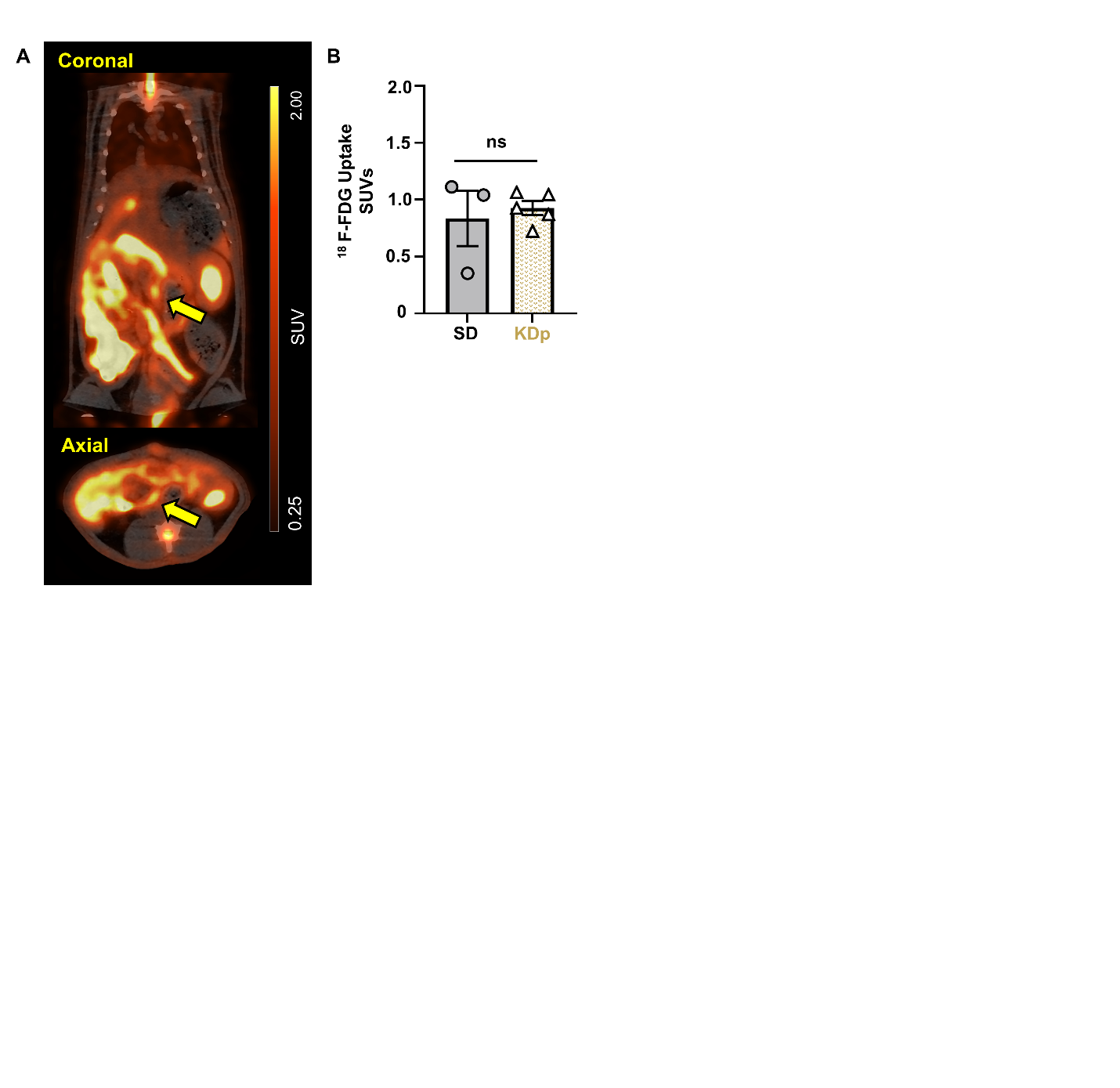


**Supplementary Figure S4: F- fluorodeoxyglucose PET/CT uptake quantification at week 1.**

(**A**) Representative PET/CT coronal and axial images showing the AAA accumulation of ^18^F-FDG (yellow arrow). (**B**) Uptake quantification for SD and KDp (SUV=0.83±0.4 and 0.92±0.1 respectively, p=ns).

**
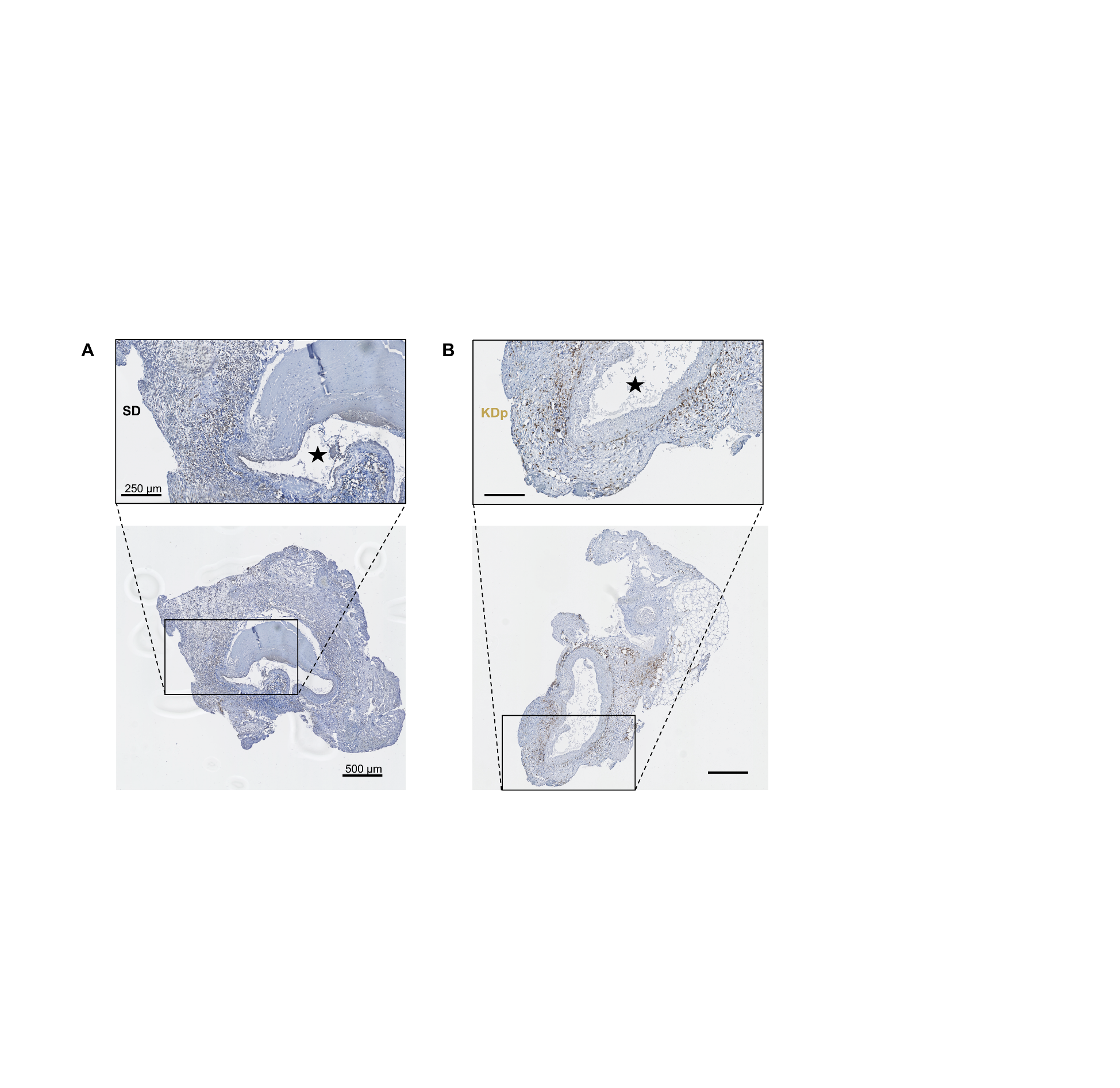
**

Supplementary Figure S5: CD68 + Cells (DAB) in SD and KDp AAA wall. (A) SD AAA tissue harvested at day 6 (week 1) with CD68 staining (DAB) at 10x and 5x magnification respectively. (B) KD AAA tissue harvested at day 6 (week 1) with CD68 staining (DAB) at 10x and 5x magnification respectively.

**
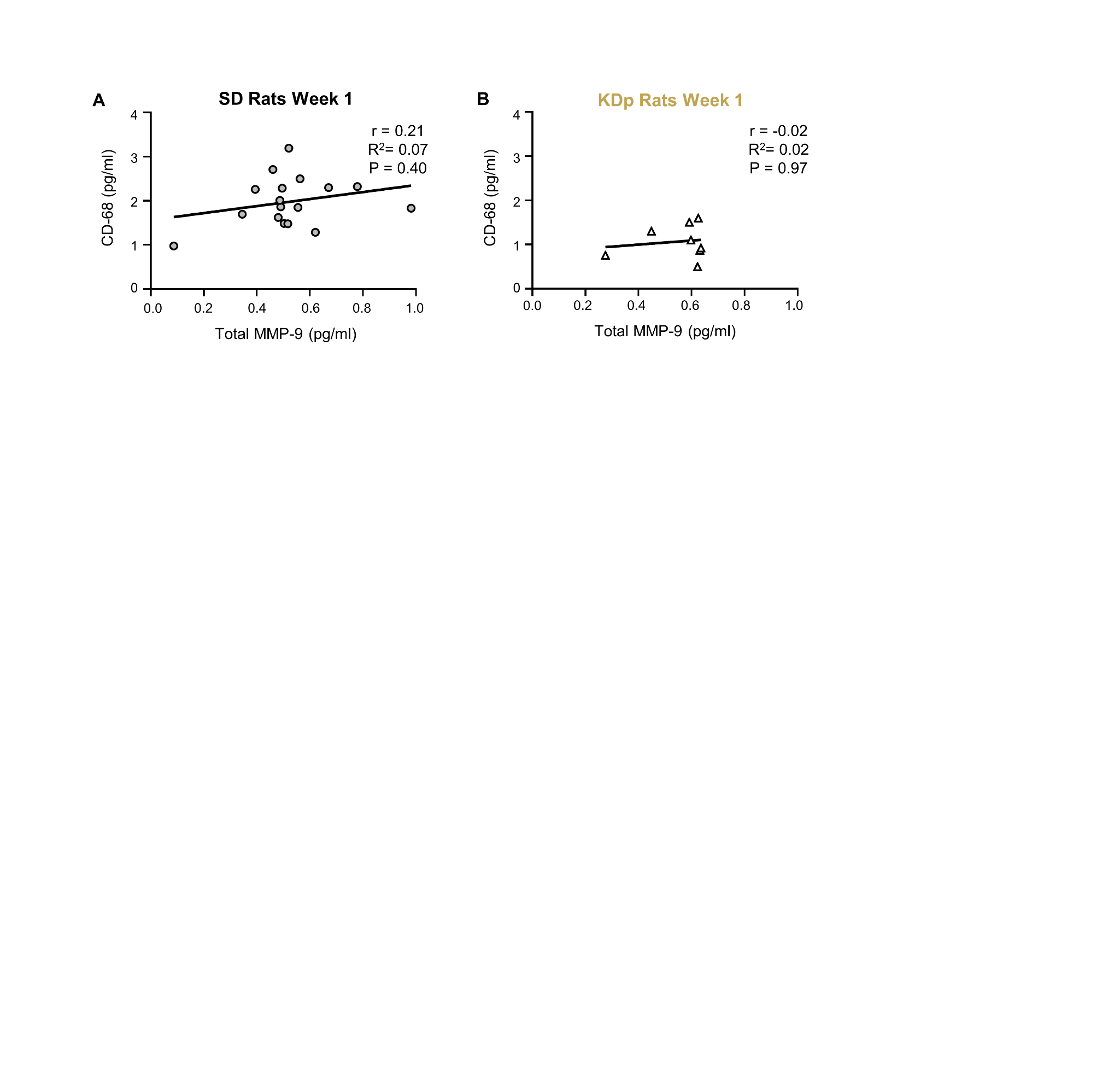
**

**Supplementary Figure S6: CD-68 and MMP-9 correlations in AAA tissue of SD and KDp rats.** (**A**) No correlation was observed between CD68 and MMP9 from ELISA analysis in SD

rats (**B**) No correlation was observed between CD68 and MMP9 from ELISA analysis in KDp rats.


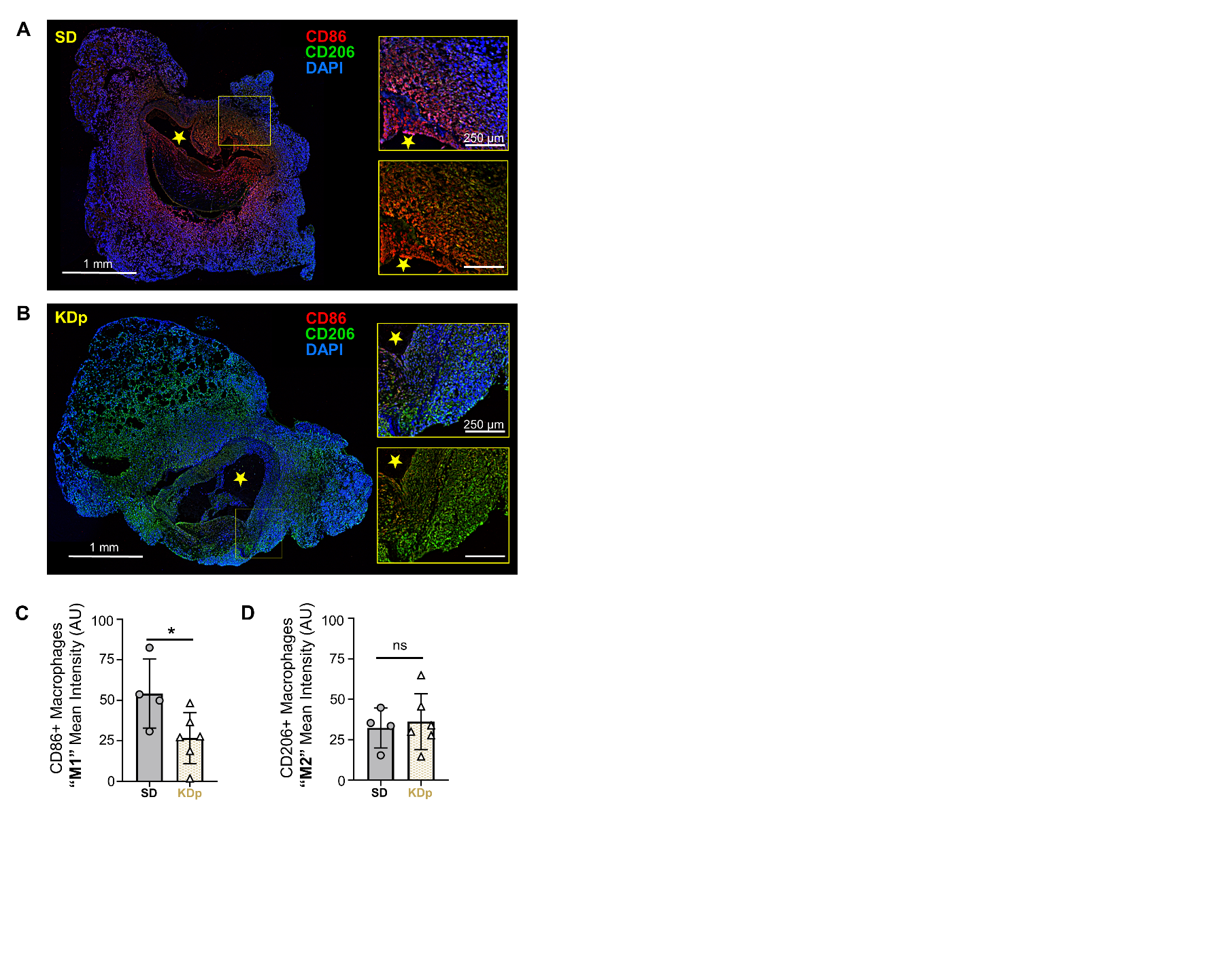


**Supplementary Figure S7**: **Macrphage phenotype population in AAA.** (**A** & **B**) Immunofluorescense staining of abdomional aortas (cross-sectional; 5c magnification and 10x magnification) marked with CD86 (in red: CD68+ M1 or pro-inflammatory macrophages) and CD206 marker (in green: CD206+ M2 or anti-inflammatory macrophages) to visualize macrophage population phenotype infiltration within the AAA of SD and KDp respectively. Yellow star indicates aortic lumen. (**C**) M1 macrophage marker CD86 content at week 1 in AAA tissue of SD and KDp rats (22±8 vs 10±6 respectively; p=0.04). (**D**) M2 macrophage marker CD206 content at week 1 in AAA tissue of SD and KDp rats (13±5 vs 14±7 respectively; p=ns).


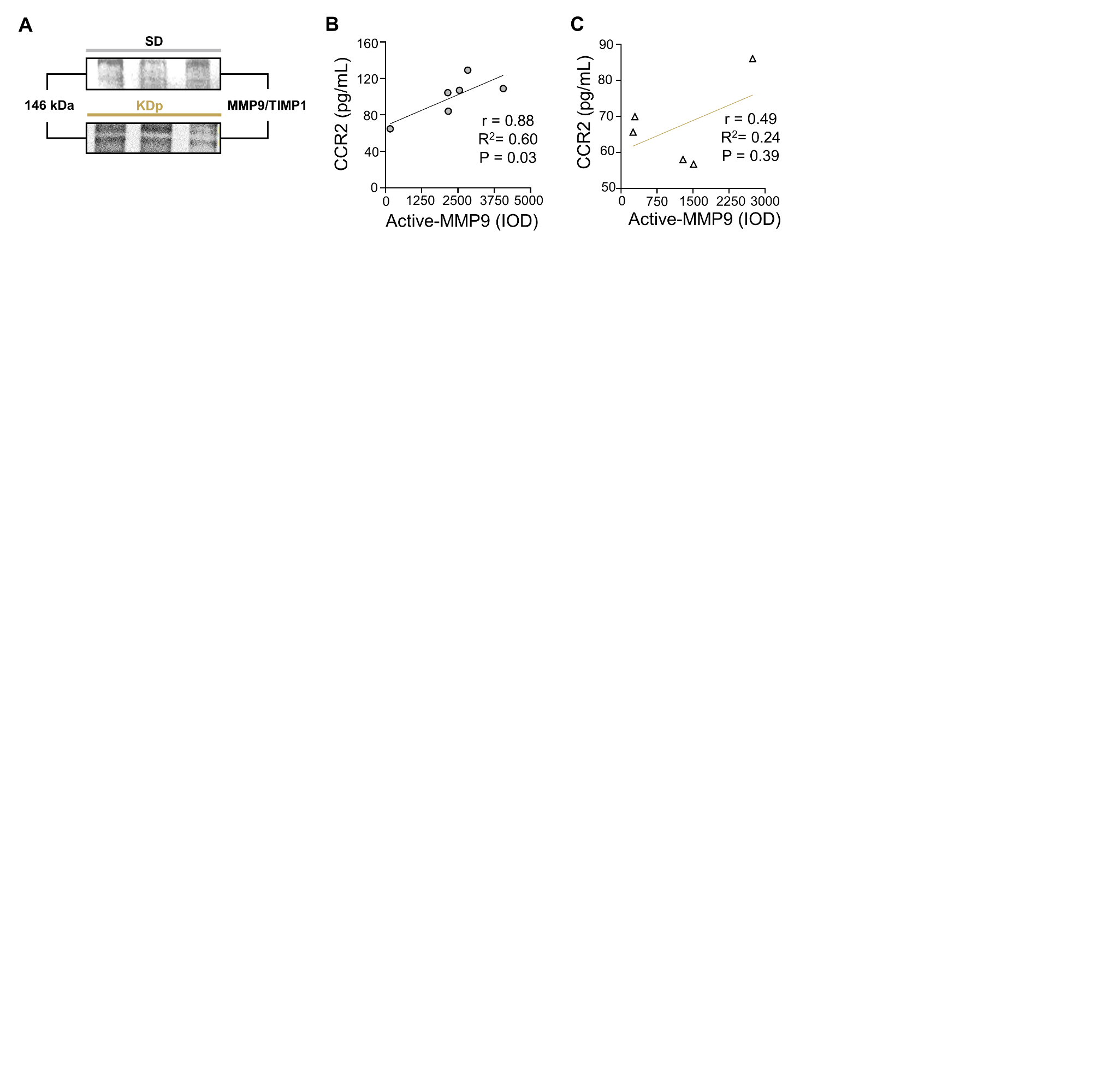


**Supplementary Figure S8: MMP9/TIMP1 and MMP/CCR2 correlation analysis** (**A**) Zymography demonstrating MMP9/TIMP1 complex levels in AAA tissue of SD and KDp rats (**B** & **C**) Positive correlation between CCR2 and active MMP9 in SD and KDp, respectively.


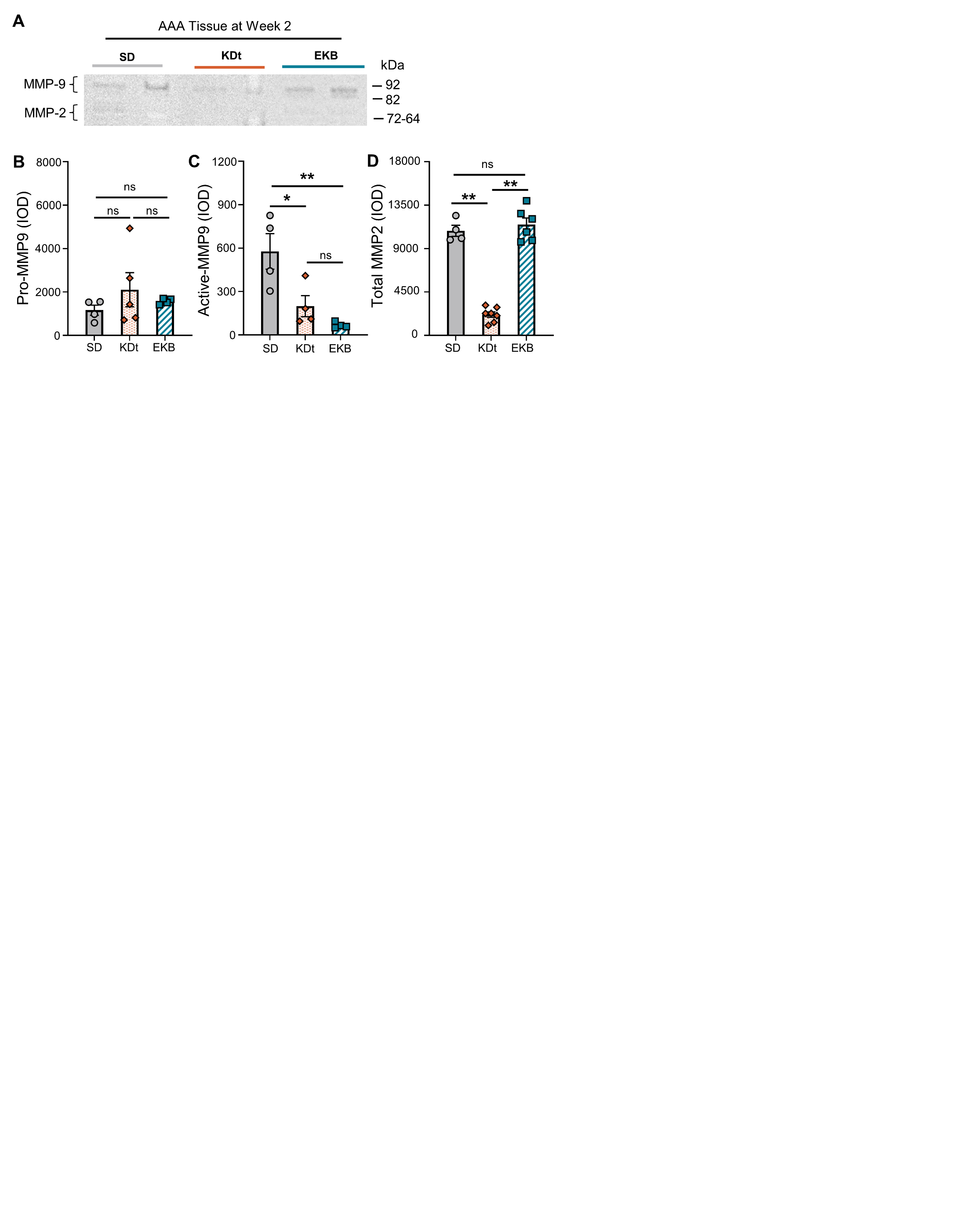


**Supplementary Figure S9: Impact of therapeutic ketosis on MMP balance.** **(A)** Representative zymogram gels of AAA tissue homogenates at week 2, demonstrating pro- and active MMP-9 and total MMP-2 in rats maintained on SD, KDt, and EKB. (**B**) Pro MMP9 levels for SD vs KDt (1.2±0.5 x10^3^ and 2.1±1.7 x10^3^; p = ns), and SD vs EKB (1.6±0.12 x10^3^; p = ns). (**C**) Active MMP9 levels for SD vs KDt (5.8±2.4 x10^2^ and 2±1.4 x10^2^; p = 0.02), and SD vs EKB (0.7±0.2 x10^2^; p = 0.005). (**D**) Total MMP-2 levels for SD vs KDt (10.8±1.1 x10^3^ and 2.1±0.7 x10^3^; p < 0.001), and SD vs EKB (11.5±1.7 x10^3^; p = ns). Pro and active MMP9 and total MMP2 levels were measured by integrated optical density (IOD) in AAA tissue, and analyzed using one-way ANOVA. (**E**) Data presented as mean ± standard deviation. ns > 0.05, *p < 0.05, **p < 0.01, ***p < 0.001 using ordinary one-way ANOVA or two-way ANOVA with multiple comparisons.


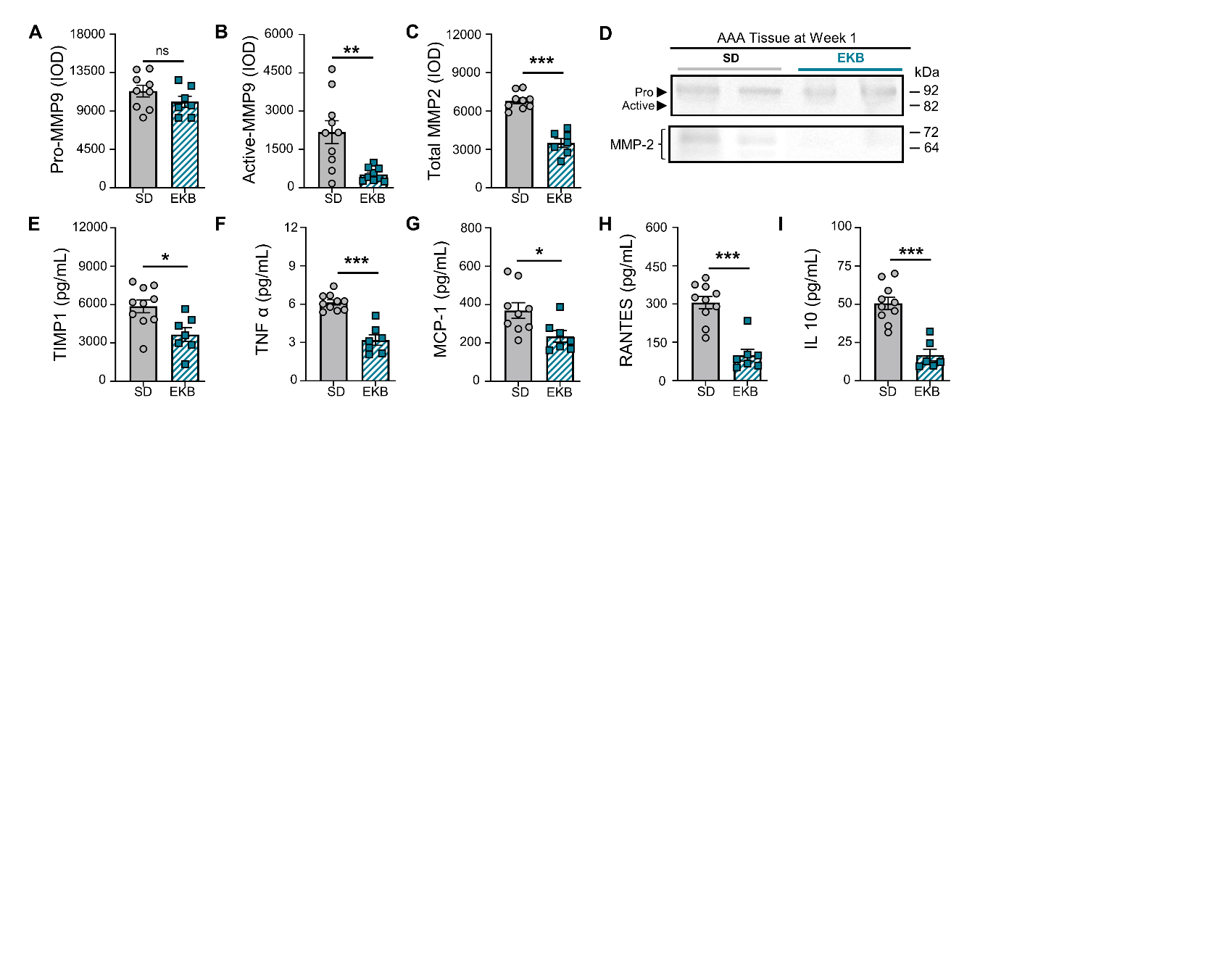


**Supplementary Figure S10: Impact of EKB Supplements on Zymography and Inflammatory Cytokines at Week 1. (A)** Pro MMP9 levels for SD (11.3±2 x10^3^) vs EKB rats (10±1.7 x10^3^; p=ns) (**B)** Active MMP9 levels for SD (2.2±1.4 x10^3^) vs EKB rats (0.5±0.3 x10^3^; p=0.01) **(C)** Total MMP2 levels for SD (6.8±0.7 x10^3^) vs EKB rats (3.5±0.9 x10^3^; p<0.001). Pro and active MMP9 and total MMP2 levels were measured by integrated optical density (IOD) in AAA tissue harvested at week 1. **(D)** Representative zymogram from AAA tissue homogenates at week 1 demonstrating pro and active MMP9 and total MMP2 levels in SD and EKB rats. ELISA of AAA tissue homogenates in SD vs EKB rats provided levels of **(E)**, TIMP 1 (5.9±1.6 x10^3^ vs 3.6±1.4 x10^3^ respectively; p=0.01) **(F)**, Pro-inflammatory marker TNFα (6.1±0.6 vs 3.2±1respectively; p=0.001) **(G & H)**, chemokines MCP 1 (3.7±1.2 x10^2^ vs 2.3±0.8 x10^2^ respectively; p=0.02) and RANTES (3±0.7 x10^2^ vs 1±0.6 x10^2^ respectively; p<0.001) and **(I)**, anti-inflammatory cytokine IL-10 (50±13 vs 17±9 respectively; p<0.001) Data presented as mean ± standard deviation. ns > 0.05, *p < 0.05, **p < 0.01, ***p < 0.001 using Student’s t test.


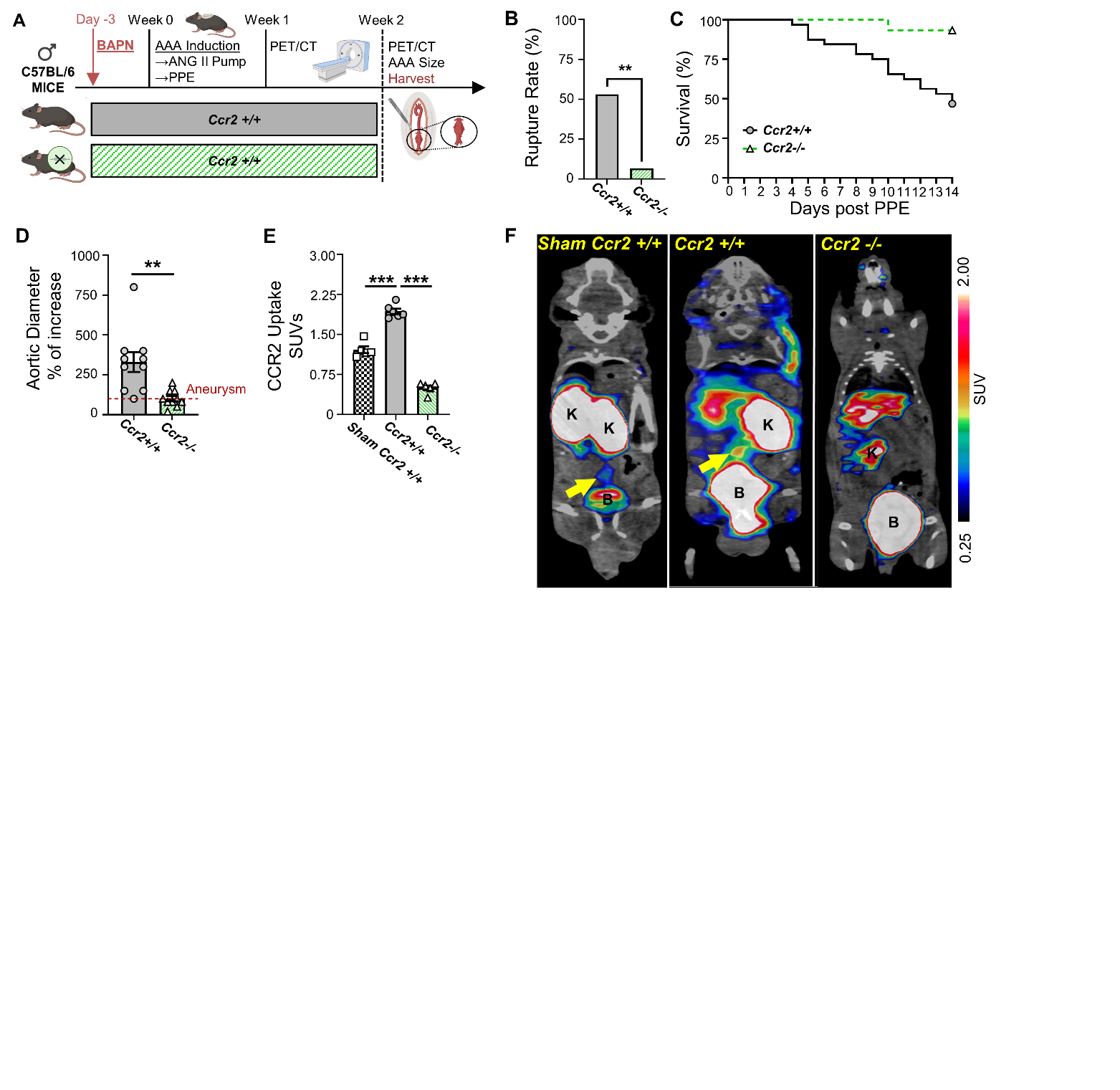


Supplementary Figure S11: *Ccr2*-/- mice have reduced AAA formation and incidence of rupture. (A) Adult male *Ccr2+/+* and *Ccr2-/-* mice underwent open aortic exposure and administration of PPE along with implantation of subcutenous ANG II osmotic pump to induce formation of AAAs. Mice also received β-aminoprionitrile (BAPN) to promote AAA rupture. (B) Incidence of AAA rupture was higher in *Ccr2+/+* mice compared to *Ccr2-/-* mice (53%, 17/32 vs 6%, 1/15; p=0.003). (C) Kaplan-Meier curve demonstrated higher survival rate in *Ccr2-/-* mice. (D) Compared to *Ccr2+/+* mice, *Ccr2-/-* mice had significantly reduced aortic diameters at week 2 (330±197 vs 101 ± 53; p = 0.002). (E) Quantitative tracer uptake of CCR2 content in AAA tissue for *Ccr2+/+ (*1.9 ± 0.1) vs sham *Ccr2+/+* (1.21± 0.1; p < 0.001) and *Ccr2-/-* (0.5 ± 0.1; p < 0.001) at week 1. (F) Representative PET/CT coronal images at week 1 post-PPE exposure demonstrating higher CCR2 content in AAA tissue (yellow arrow) of *Ccr2+/+* mice compared to sham and *Ccr2-/-* controls (K: kidney, B: Bladder).


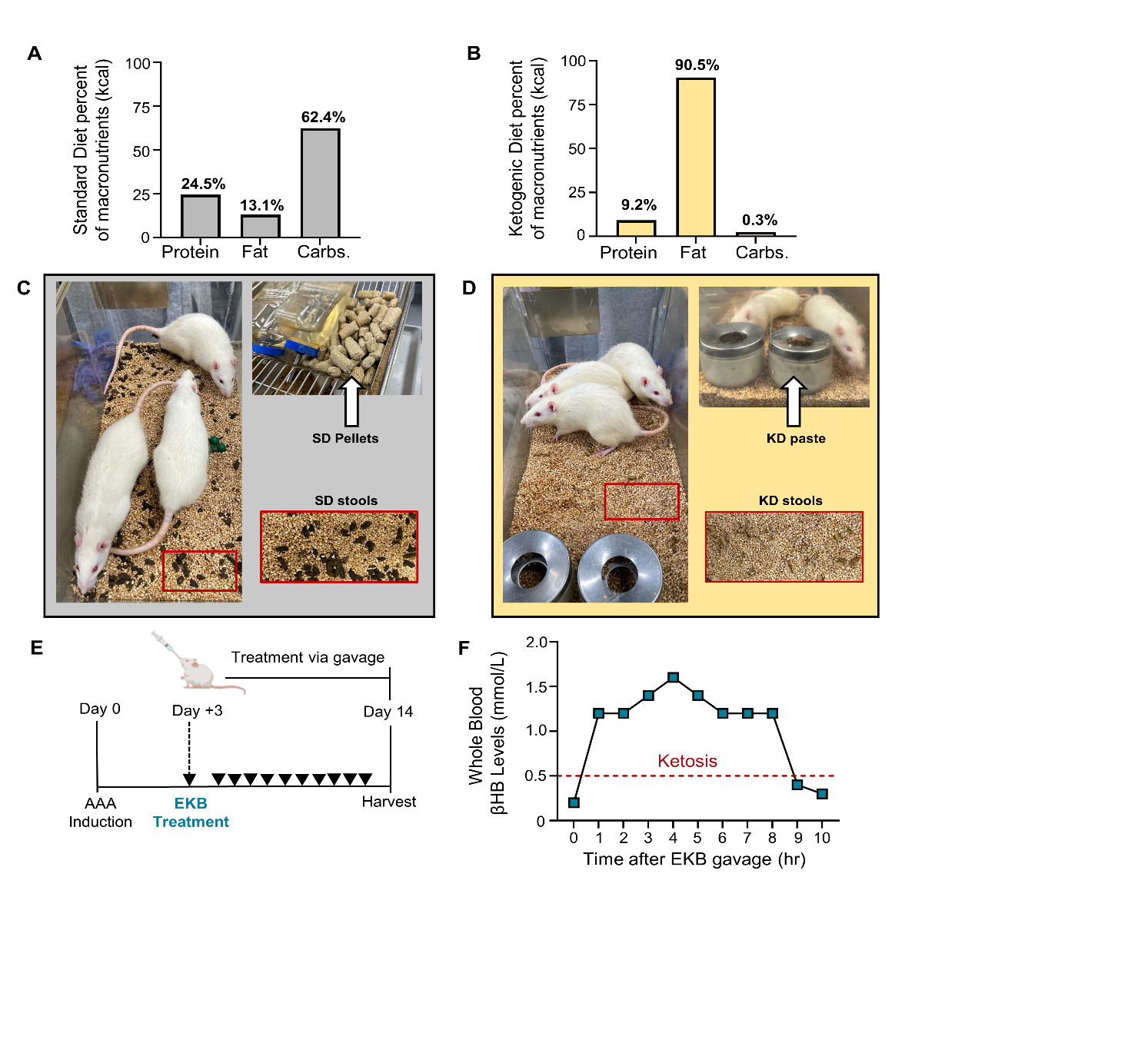


Supplementary Figure S12: Animal Diet Composition and Interventions. (A) Standard chow diet (SD) source of calories expressed in percentages. (B) Ketogenic diet (KD) source of calories expressed in percentages. (C) Appearance of SD diet and rat stool characteristics. (D) Appearance of KD diet and stool characteristics. (E) Treatment timeline with EKB via gavage. (F) Ketosis (βHB whole blood levels > 0.5 mM/L) verification in EKB rats 10 hours after gavage.


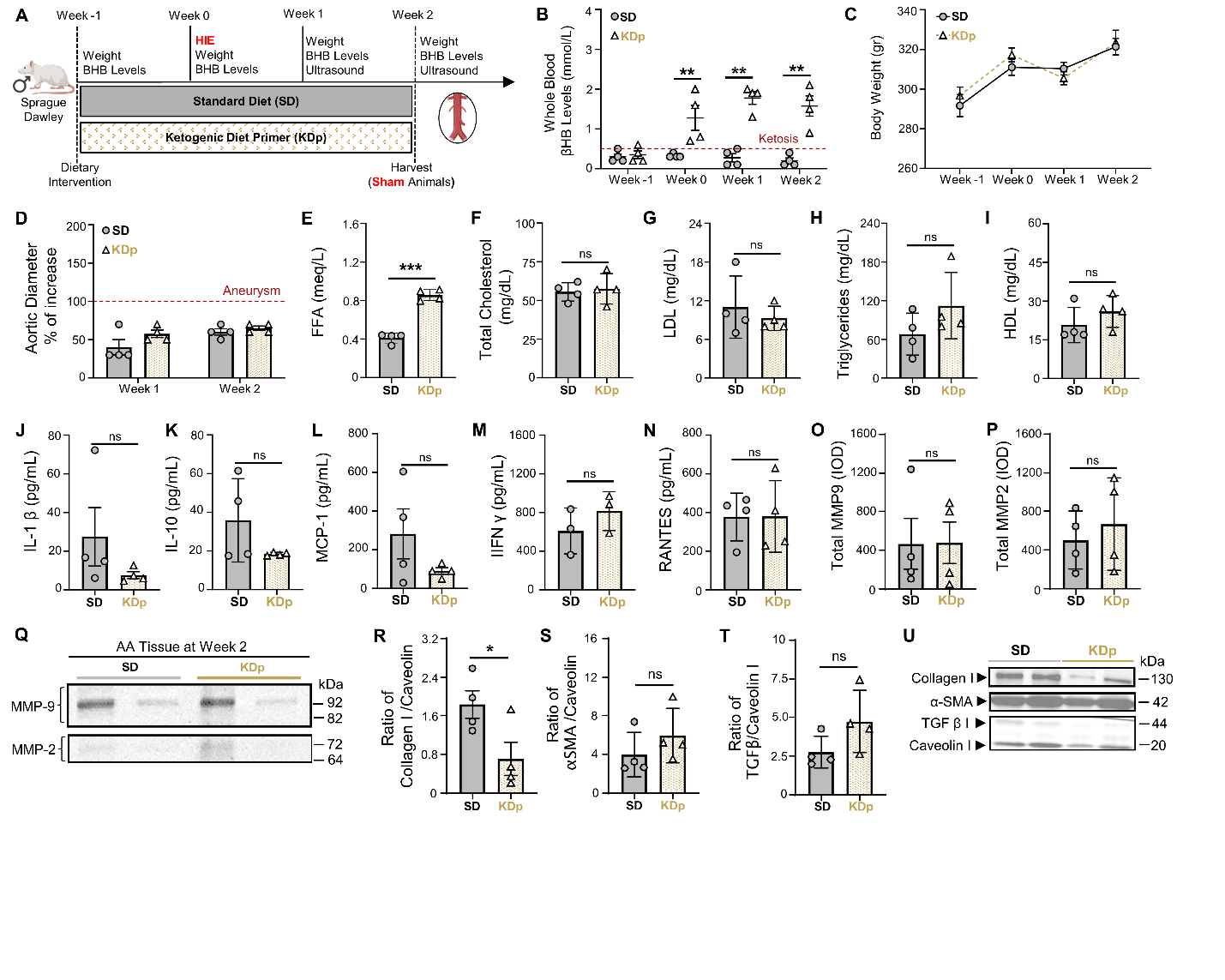


Supplementary Figure S13. Ketosis effect on HIE Sham Contrls. (A) Rats underwent *exposure* to HIE for Sham-control model. The experimental group was given a ketogenic diet that started one-week prior to HIE exposure (KDp; N=4) while the control group was fed a standard chow diet (SD; N=4). (B) Ketosis (βHB whole blood levels > 0.5 mM/L) was verified at week -1, 0, 1 and 2 in SD (0.3±0.1, 0.3±0.05, 0.3±0.2 and 0.2±0.1) and KDp rats (0.3±02, 1.2±0.6, 1.7±0.3 and 1.5±0.5) respectively (p < 0.01). (C) Body weight in SD vs KDp rats at week -1 (287±13 vs 297±8), week 0 (305±12 vs 317±7), week 1 (295±31 vs 305±7) and at week 2 (310±23 vs 323±12) respectively (p = ns). (D) Percent aortic diameter in SD vs KDp rats at week 1 (40±20 vs 57±10; p = ns) and at week 2 (60±8 vs 65±6; p = ns) respectively (aneurysms were defined by a >100% increase in the aortic diameter compared with pretreatment measurements). (E) Free fatty Acids (FFA) serum levels in meq/L in rats fed SD vs KDp (0.4±0.05 vs 0.86±0.06 p < 0.001). (F) Total cholesterol (TC) serum levels (mg/dL) in rats fed SD vs KDp (55±6 vs 57±10; p = ns). (G) Low-density lipoprotein (LDL) serum levels (mg/dL) in rats fed SD vs KDp (11±5 vs 9±2; p = ns). (H) Triglycerides (TG) serum levels (mg/dL) in rats fed SD vs KDp (68±32 vs 112±51; p = ns). (I) High-density lipoprotein (HDL) serum levels in mg/dL in rats fed SD vs KDp (21±7 vs 26±6; p = ns). ELISA analysis for SD vs KDp rats (J) IL 1β content (27±30 vs 8±3), (K) IL-10 content (36±21 vs 18±0.8), (L) Chemokine MCP-1 content (282±258 vs 90±33), (M) IFNγ content (609±237 vs 814±200) and (N) RANTES content (377±123 vs 380±185). (O) Total MMP9 levels at week 2 in AAA tissue of SD and KDp rats (2.8±3x10^3^ vs 2.9±2.7x10^3^ respectively; p= ns). (P) Total MMP2 leveles at week 2 in AAA tissue of SD and KDp rats (0.5±0.3x10^3^ vs 0.7±0.5x10^3^ respectively; p = ns). (Q) Representative zymogram from AAA itssue homogenates at week 2 demonstarting total MMP-9 and MMP-2 levels in SD and KDp rats. (R) Collagen I protein content expressed as a ratio to Caveolin 1 content in AAA tissue of SD and KDp rats (1.8±0.6 vs 0.7±0.6 respectively; p = 0.04). (S) α-SMA protein content expressed as a ratio to Caveolin 1 content in AAA tissue of SD and KDp rats (3.9±2.2 vs 5.9±2.8 respectively; p = ns). (T) TGFβ protein content expressed as a ratio to Caveolin 1 conten in AAA tissue of SD and KDp rats (2.7±1 vs 4.7±2 respectively; p = ns). (U) Representative western blots of collagen 1, α-SMA, TGFβ-1 and Caveolin 1 in AAA tissue. Data presented as mean ± standard deviation. ns > 0.05, *p < 0.05, **p < 0.01, ***p < 0.001 using Student’s t test.


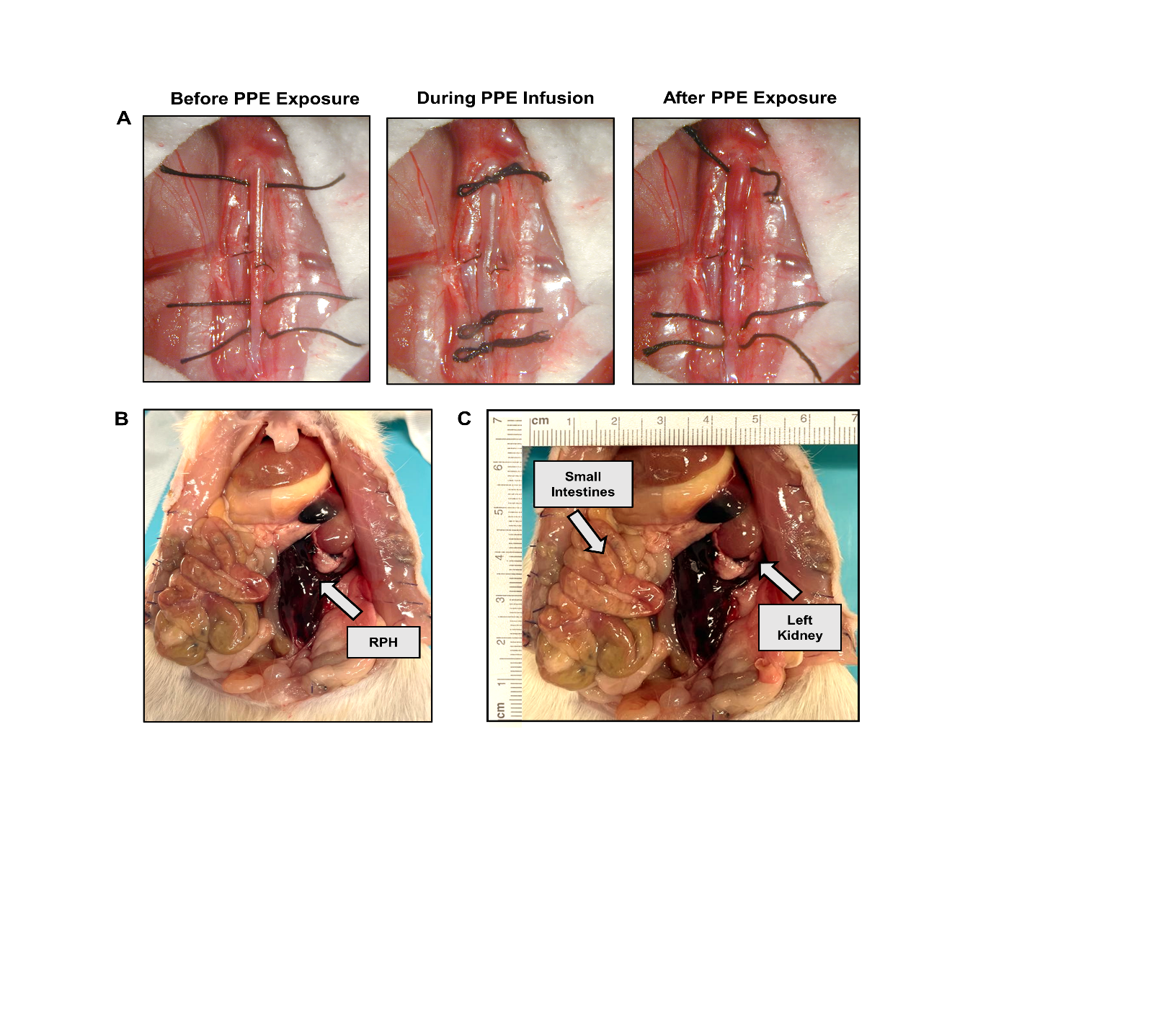


Supplementary Figure S14: Exposure of the Abdominal Aorta to PPE, and AAA Rupture Findings. (A) Exposure of the abdominal aorta for AAA induction using PPE, before aortotomy and PPE infusion, during PPE infusion and aortic dilation, and release of the temporary proximal and distal control sutures (B) Representative AAA rupture into the left retroperitoneum and the associated retroperitoneal hematoma (RPH) identified with arrow. (C) Anatomic references around hematoma expansion. The RPH extension in this animal was approximately 4 x 2 cm.
